# A catchment-scale dataset of soil properties and their mid-infrared spectra

**DOI:** 10.1016/j.dib.2025.111510

**Published:** 2025-03-26

**Authors:** Alexandre M.J.-C. Wadoux, Felix Stumpf, Thomas Scholten

**Affiliations:** a LISAH, Univ. Montpellier, AgroParisTech, INRAE, IRD, L'Institut Agro, Montpellier, France; b Swiss Compentence Center for Soil (KOBO), Zollikofen, Switzerland; c Department of Geosciences, Chair of Soil Science and Geomorphology, University of Tübingen, Tübingen, Germany

**Keywords:** Erosion, Soil function, Digital soil mapping, Three-gorges Dam, Soil health

## Abstract

The dataset presents information on soil properties and their associated mid-infrared spectra for a drainage basin of 4.2 km^2^, referred to as Upper Badong catchment (31°1′24′′N, 110°20′35′′E) in the Hubei province, China. Data were collected for topsoil in a highly diverse terrace catchment composed of woodland, cropland and small farm building. Soil properties included in this dataset are pH, texture (i.e. clay, silt and sand content), total carbon, organic carbon and CaCO_3_. In addition, the soil samples were scanned in the mid-infrared range. The data collection processed involved three field campaigns during 2013 and 2014 where topsoil samples were collected in a standardized way across all sites, and soil analyses in the laboratory of soil science following standard procedures. The dataset offers insights into the spatial variation of soil properties in a highly diverse catchment of central China. Researchers interested in soil research can use this dataset for various purposes, including building digital soil mapping models or soil spectroscopic models, benchmarking of models with other datasets, and research in soil erosion modelling.

Specifications Table

**Table:** 

Subject	Agricultural Sciences (Soil Science), Environmental Science (Ecology), Data Science
Specific subject area	Soil properties; Soil health indicators; Soil survey
Type of data	Tables (.txt format) Additional simplified variables created by consolidating raw data.
Data collection	Data were collected during three soil surveys in 2013–2014 within the framework of the Sino-German YangtzeGeo project. The soil samples were analysed in the Laboratory for Soil Science and Geoecology at Tübingen University using standard laboratory procedures. The mid-infrared spectra were obtained with a Vertex 70 Fourier transform infrared spectrometer (Bruker Optics Inc.). The specific laboratory procedures used for soil analyses are documents in the methods.
Data source location	The data were collected in the upper Badong catchment, Hubei province, China, located 74 km upstream the Three-Gorges Dam on the Yangtze River. The catchment is centred at coordinates 31°1′24′′N and 110°20′35′′E.
Data accessibility	Repository name: Zenodo Data identification number: 10.5281/zenodo.14557348 Direct URL to data: https://zenodo.org/records/14557348
Related research article	Stumpf, F., Goebes, P., Schmidt, K., Schindewolf, M., Schönbrodt‐Stitt, S., Wadoux, A., Xiang, W & Scholten, T. (2017). Sediment reallocations due to erosive rainfall events in the Three Gorges Reservoir Area, Central China. Land degradation & development, 28(4), 1212–1227.

## 1 Value of the Data

• The data can be used for research on soil spatial modelling and soil spectroscopy, and to determine how terrain and other environmental variables affect the pattern of soil properties.
• The dataset can be integrated with other datasets derived from remote sensing and hydrological measurement to investigate the dynamic of soil erosion in a highly diverse cultivated drainage basin.
• Our dataset is valuable to research in benchmarking the model accuracy of digital soil mapping or spectroscopic models.
• The dataset can be integrated with other regional and global dataset on soil properties to improve national and global soil mapping efforts.

## 2 Background

Spatial modelling of soil properties using digital soil mapping techniques and soil spectroscopy analyses with mid-infrared spectroscopy are popular research fields of soil science, both for methodological development (e.g. in sampling design optimization or geostatistical model development) and to learn about basic soil processes (e.g. through analysis of the infrared spectra). This dataset is a compilation of three soil surveys realized within the Sino-German YangtzeGeo project in 2013–2014. The study area is a catchment North of the city of Badong, in the Hubei province, China. The spatial dataset reports on the collection of soil samples which were analyzed using conventional laboratory methods. This dataset was extensively used for research purposes on soil sampling design optimization [2], soil erosion [3], infrared spectroscopy [5] and uncertainty guided sampling to improve digital soil mapping [4].

## 3 Data Description

The dataset comprises two text files stored in the Zenodo repository.

**Dataset *soil_data.txt*** – Matrix or X rows and X columns where each row is a unique soil sample with two unique identifiers, the sampling coordinates x and y, and the list of soil properties CaCO_3_, pH, total carbon, organic carbon as well as texture divided into coarse, medium, fine, very fine and total sand, coarse, medium, fine and total silt, and total clay contents Table 1.

**Table 1:** Summary statistics of the soil properties included in the dataset.

Property	Minimum	1st quartile	Median	Mean	3rd quartile	Maximum
CaCO_3_ (%)	0	0.4	0.6	1.5	0.8	23.6
Total carbon (%)	0.7	1.4	1.8	2.17	2.5	7.4
pH (unitless)	0	5.85	6.7	6.55	7.4	8.3
Organic carbon (%)	0.7	1.3	1.6	2.03	2.4	7.4
Total sand (%)	0.68	2.46	3.19	7.65	10.61	43.88
Total silt (%)	35.20	56.71	63.07	61.06	67.08	76.28
Total clay (%)	16.79	26.57	30.99	31.27	35.69	51

**Fig. 1:**
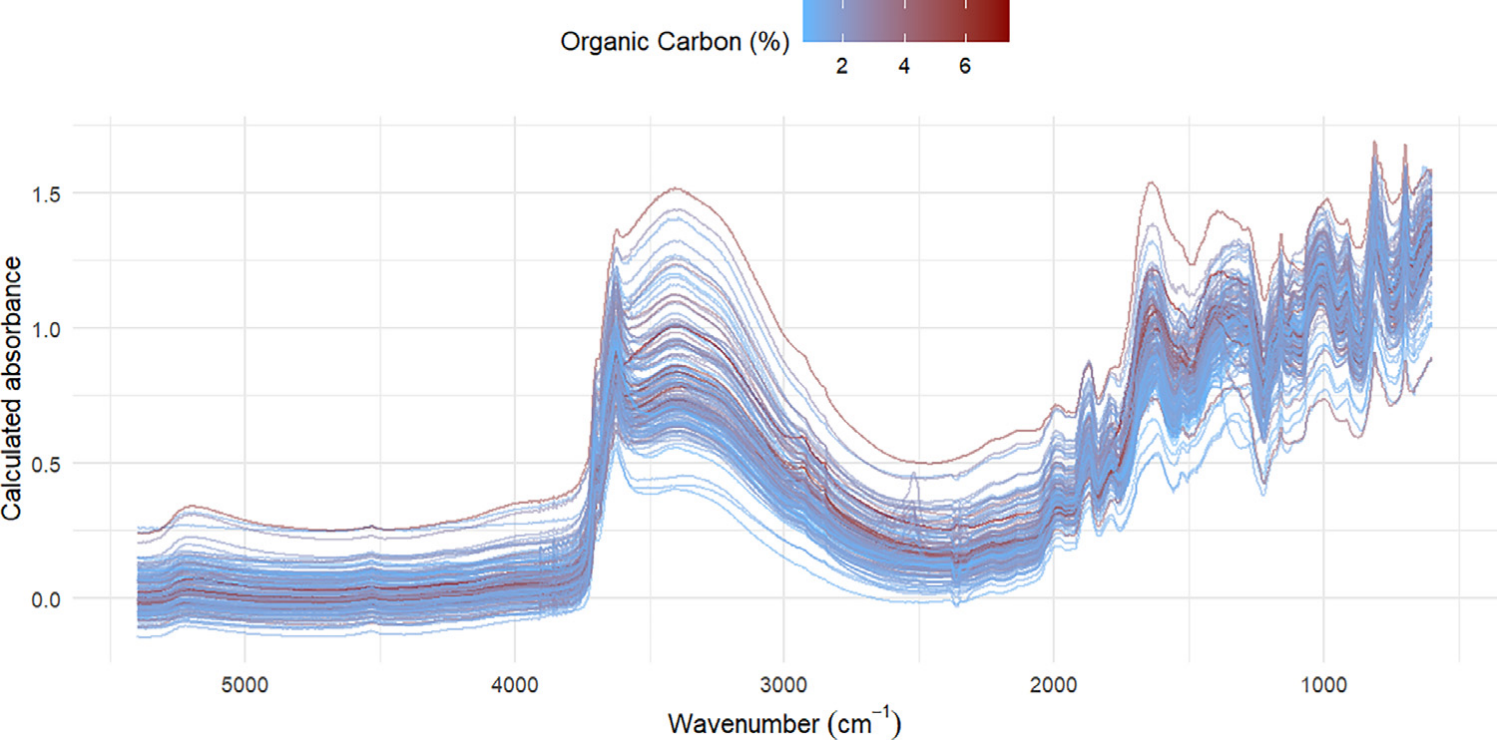
Mid-infrared spectra of the soil samples. The colour represents the percent of organic carbon associated to the spectrum. (For interpretation of the references to colour in this figure legend, the reader is referred to the web version of this article.)

**Dataset *MID_IR_spectra.txt***: Matrix of X rows and X columns, where each row is a unique soil sample with the same two unique identifiers than *soil_data.txt*. Each column is then the wavenumber in cm^-1^Fig. 1.

## 4 Experimental Design, Materials and Methods

### 4.1 Data collection

In October 2012, 55 topsoil samples were collected in a preliminary reconnaissance survey using stratified random sampling. A second field campaign was organized in June 2013 where an additional 25 samples were taken. In two additional campaigns in May 2014 and November 2014, another 59 samples were collected using conditioned Latin hypercube sample. All 139 samples were obtained and analysed identically. At each sampling site, composite samples were collected by pooling five topsoil (0–25 cm depth) sub-samples: four from the corners of a 40 cm × 40 cm square and one from its centre. The 5 samples were mixed, reduced to approximately 500 g and placed into labelled plastic bags. At each sampling site, the coordinates were taken using a Garmin GPSMAP 67 handheld GPS system expressed in EPSG:32649.

### 4.2 Soil property analyses

All samples were oven-dried at 40 °C during 24h

Soil pH was measured with bi-distilled H_2_O using a soil solution ratio of 1:2.5 (WTW pH-meter with Sentix81 electrodes, Weilheim, Germany).

The proportional fractions of coarse sand (CS: 2–0.63 mm), medium sand (MS: 0.63–0.2 mm), and fine sand (FS: 0.2–0.063 mm) were determined according to DIN ISO 11277:2002-08 (2002). Samples were oven-dried at 105 °C, ground, and dispersed into primary particles using Na₄P₂O₇ in suspension, then sieved to separate the sand fractions (Müller et al., 2009). Additionally, particles smaller than 0.63 mm were sieved to isolate sand content, while silt and clay fractions were separated using the Sedigraph III 5120 by Micromeritics GmbH. The equivalent diameters of the soil particles in the coarse silt fraction range from 0.063 to 0.02 mm, in the medium silt fraction from 0.02 to 0.0063 mm and the fine silt fraction from 0.0063 to 0.002. If the total content of all fractions deviated by more than 5 % from the ideal 100 %, the analysis was repeated.

We used aliquots (50 g) of the homogenized and dried (40 °C) composite samples to determine the soil organic carbon content. The soil organic matter (SOM) was calculated as a function of carbonate (CaCO₃) content and total carbon estimation. Carbonate content was determined using the Scheibler method, which measures the volumetric carbonates through the CO₂ emitted during the reaction of carbonates with hydrochloric acid in a calcimeter (Eijkelkamp, Giesbeek). Total carbon content was estimated using an element analyzer (vario EL III, Elementar Analysesysteme GmbH, Germany, in CNS mode) with helium atmosphere and oxidative heat combustion at 1150 °C was used for the measurement of total carbon (TC). The difference of total inorganic carbon and TC was used for the calculation of the soil organic carbon (SOC) content [1].

### 4.3 Soil infrared spectroscopy

Each sample was air-dried, sieved to 2 mm, and ball-milled to prepare for laboratory analysis, excluding plant material and stones. Background measurements were performed initially with an empty sample compartment using 32 co-added repetitions. Soil samples were placed in sample cups measuring 0.5 cm in diameter and 0.5 cm in depth, with the surface smoothed using a spatula to maximize light reflection and minimize the signal-to-noise ratio.

Spectroscopic scans were conducted using a Fourier Transform Infrared (FTIR) Vertex 70 Spectrometer (Bruker, Germany), equipped with an air-cooled IR source, ZnSe optics to control air humidity, and a MIR-KBr beamsplitter covering a spectral range of 7500–370 cm⁻¹. Each sample was scanned three times in the MIR range (570–5500 cm⁻¹) for absorbance, with 64 co-added scans per measurement. Background measurements were repeated every three samples or ten minutes, and three reference samples were scanned every four hours to monitor environmental factors, such as air humidity.

After scanning, the standard deviation (SD) of the spectrum was calculated for each sample. Samples with an SD greater than 1.5 were re-scanned to ensure data quality.

## Ethics Statement

The authors read and followed the ethical requirements for publication in Data in Brief and confirmed that the current work did not involve human subjects, animal experiments, or any data collected from social media platforms.

## CREDIT Author Statement

**Alexandre Wadoux:** Data collecting, Data curation, Methodology, Writing Original Draft. **Felix Stumpf:** Data collecting, Data curation, Conceptualization, Methodology, Writing, Reviewing and Editing. **Thomas Scholten:** Funding Acquisition, Conceptualization, Methodology, Writing, Reviewing and Editing.

## CREDIT Author Statement Related Paper

Stumpf, F.: Data collecting, Data curation, Conceptualization, Methodology, Writing, Original Draft. Goebes, P.: Data collecting, Data curation, Conceptualization, Methodology, Writing, Reviewing and Editing. Schmidt, K.: Data collecting, Data curation, Conceptualization, Methodology, Writing, Reviewing and Editing. Schindewolf, M.: Data collecting, Data curation, Conceptualization, Methodology, Writing, Reviewing and Editing. Schönbrodt‐Stitt, S.: Data collecting, Data curation, Conceptualization, Methodology, Writing, Reviewing and Editing. Wadoux, A.: Data collecting, Data curation, Conceptualization, Methodology, Writing, Reviewing and Editing. Xiang, W: Data collecting, Data curation, Conceptualization, Methodology, Writing, Reviewing and Editing. Scholten, T.: Funding Acquisition, Conceptualization, Methodology, Writing, Reviewing and Editing.

## Data Availability

ZenodoA catchment-scale dataset of soil properties and their mid-infrared spectra (Original data).

## Appendix Supplementary materials

Image, application 1
