# Supplementary material for: A catchment-scale dataset of soil properties and their mid-infrared spectra
Source: Data Brief. 2025 Mar 26;60:111510. doi: 10.1016/j.dib.2025.111510 (PMC11999479; doi:10.1016/j.dib.2025.111510)
Supplement: Supplementary file 1 [file mmc1.pdf]

LAND DEGRADATION & DEVELOPMENT
Land Degrad. Develop. **28**: 1212–1227 (2017)
Published online 24 February 2016 in Wiley Online Library (wileyonlinelibrary.com) **DOI**: 10.1002/ldr.2503

SEDIMENT REALLOCATIONS DUE TO EROSIVE RAINFALL EVENTS IN THE THREE GORGES RESERVOIR AREA, CENTRAL CHINA

Felix Stumpf[1]*, Philipp Goebes[1], Karsten Schmidt[1], Marcus Schindewolf[2], Sarah Schönbrodt-Stitt[1], Alexandre Wadoux[3], Wei Xiang[4], Thomas Scholten[1]

[1]*Department of Geosciences, Chair of Soil Science and Geomorphology, University of Tübingen, Tübingen, Germany*
[2]*Soil and Water Conservation Unit, Technical University Freiberg, Freiberg, Germany*
[3]*University & Research Centre, Wageningen UR, Wageningen, The Netherlands*
[4]*Department of Geotechnical Engineering and Engineering Technology, China University of Geosciences Wuhan, Wuhan, PR China*

Received: 14 October 2015; Revised: 14 January 2016; Accepted: 17 January 2016

ABSTRACT

Soil erosion by water outlines a major threat to the Three Gorges Reservoir Area in China. A detailed assessment of soil conservation measures requires a tool that spatially identifies sediment reallocations due to rainfall–runoff events in catchments. We applied EROSION 3D as a physically based soil erosion and deposition model in a small mountainous catchment. Generally, we aim to provide a methodological frame that facilitates the model parametrization in a data scarce environment and to identify sediment sources and deposits. We used digital soil mapping techniques to generate spatially distributed soil property information for parametrization. For model calibration and validation, we continuously monitored the catchment on rainfall, runoff and sediment yield for a period of 12 months. The model performed well for large events (sediment yield > 1 Mg) with an averaged individual model error of 7.5%, while small events showed an average error of 36.2%. We focused on the large events to evaluate reallocation patterns. Erosion occurred in 11.1% of the study area with an average erosion rate of 49.9 Mg ha^{-1}. Erosion mainly occurred on crop rotation areas with a spatial proportion of 69.2% for 'corn-rapeseed' and 69.1% for 'potato-cabbage'. Deposition occurred on 11.0%. Forested areas (9.7%), infrastructure (41.0%), cropland (corn-rapeseed: 13.6%, potato-cabbage: 11.3%) and grassland (18.4%) were affected by deposition. Because the vast majority of annual sediment yields (80.3%) were associated to a few large erosive events, the modelling approach provides a useful tool to spatially assess soil erosion control and conservation measures. Copyright © 2016 John Wiley & Sons, Ltd.

KEY WORDS: sediment sources and deposits; EROSION 3D-model; rainfall–runoff event; catchment scale; Three Gorges Reservoir Area

INTRODUCTION

Soils are the foundation of all terrestrial ecosystems and develop at the intersection of atmosphere, biosphere, hydrosphere and lithosphere (Brevik *et al.,* 2015). In this context, soils inhere ecosystem functions that have direct impact on human societies (Berendse *et al.,* 2015). Examples of these soil functions include water purification by filtration, food production by forming a support system for plants, stabilizing climate change by carbon sequestration and the provision of a physical basis for human activities (Keesstra *et al.,* 2012; Goebes *et al.,* 2015). However, soil erosion and the accompanied loss of topsoil result in soil quality degradation, and therefore in a declining capacity of soils to provide the ecosystem functions (Lal, 2003; Morgan, 2005; Boardman, 2006). This implies reduced crop productivity and confined water quality due to the reallocation of nutrients and pollutants into the reservoirs (Zhao *et al.,* 2013; Ferreira *et al.,* 2015; Slimane *et al.,* 2015). Moreover, sediment depositions in reservoirs result in a declined water storage capacity and a therefore impaired water and energy supply (Palazón *et al.,* 2014; Ferreira *et al.,* 2015; Slimane *et al.,* 2015). The negative impacts of soil erosion are reinforced when adverse physio-geographic conditions, such as erosion-prone soils, steep sloping terrain and abundant rainfall, meet inappropriate agricultural practices, deforestation and construction activities (Onyando *et al.,* 2005; Park *et al.,* 2011; Wu *et al.,* 2011; Shi *et al.,* 2012; Schönbrodt-Stitt *et al.,* 2013a). Considering that rates of soil erosion exceed rates of soil formation by several orders of magnitude, soil erosion outlines a major threat to ecosystem sustainability worldwide (Verheijen *et al.,* 2009; Zhao *et al.,* 2013). Under these circumstances, soil erosion becomes potentially hazardous for human livelihood and requires erosion control measures and conservation planning (Shi *et al.,* 2004; Zhao *et al.,* 2013; Ferreira *et al.,* 2015).

Especially, the Three Gorges Reservoir Area (TGRA) in China shows high susceptibility to hazardous soil erosion because of intense anthropogenic activities and unfavourable environmental conditions (Zhang *et al.,* 2009; Schönbrodt *et al.,* 2010; Peng *et al.,* 2011; Wu *et al.,* 2011; Kepa Brian Morgan *et al.,* 2012; Shi *et al.,* 2012; Strehmel *et al.,* 2015). The TGRA covers an area of 57,802 km^2. At the maximum pool level of 175 m, the reservoir expands approximately 660 km westwards from Three Gorges Dam (TGD; Xu *et al.,* 2011). The TGD project outlines the world's

* Correspondence to: F. Stumpf, Department of Geoscience, Chair of Soil Science and Geomorphology, University of Tübingen, Rümelinstraße 19-23, Tübingen, Germany.
E-mail: felix.stumpf@uni-tuebingen.de

Copyright © 2016 John Wiley & Sons, Ltd.

largest hydroelectric scheme and was designed to increase energy supply, control seasonal floods and improve navigation on the Yangtze river (Zhang & Lou, 2011). Accompanied by the construction of TGD and the impoundment of the reservoir, 1.3 million people were resettled because of the inundation of 431 km^2 agricultural land and 35 km^2 residential areas (Xu *et al.,* 2011, 2013). About 42% of the resettlers were moved uphill to previously forested and steep mountainous sites for small scale crop cultivation (Cai *et al.,* 2005; Tan & Yao, 2006; Wu *et al.,* 2011; Zhang & Lou, 2011). In total, 96% of the TGRA exhibits mountainous and hilly terrain with steep slopes (Zhang *et al.,* 2009; Wu *et al.,* 2011; Fang *et al.,* 2013). It shows a humid subtropical climate with a unimodal rainfall regime, governed by the East-Asian monsoon. The long-term average annual precipitation is 1,146 mm, of which approximately 70% occurs from May to September (CMA, 2012). Purple soils and yellow to yellow-brown soils are dominant in the TGRA and are considered to be highly vulnerable to soil erosion (Zhang *et al.,* 2009; Peng *et al.,* 2011; Schönbrodt-Stitt *et al.,* 2013b). Estimations on the long-term annual soil losses based on empirical modelling amount to 157 million megagram (Lu & Higgitt, 2000). The soil erosion induces sedimentation of the reservoir, and hence an attenuated ecological functioning of the Yangtze basin, reduced lifespan of the dam and a declined capacity to control floods (Shi *et al.,* 2004; Zhang & Lou, 2011; Xu *et al.,* 2013). Since the 1990s, conservation measures were established to mitigate the hazardous effects. The measures include programmes to implement conservation farming practices and to stabilize steep sloping surfaces mainly by reforestation and the construction of cropland terraces (Xu *et al.,* 2013).

For a detailed assessment of the conservation measures, an efficient tool is required that spatially identifies patterns of sediment reallocations in a mountainous and highly dynamic region (Shi *et al.,* 2012). In the TGRA, the major sediment yield caused by soil erosion is attributable to only very few heavy storm events each year (Fang *et al.,* 2013). Thus, event-based estimations on sediment reallocations are of major concern for regional authorities (Peng *et al.,* 2011). Consequently, there is a demand for an event-based approach that spatially quantifies soil erosion and deposition at catchment scale (Cai *et al.,* 2005; Shi *et al.,* 2012). This is addressed by the concept of sediment connectivity, which describes transfer characteristics of sediment through a landscape system at various scales (Hooke, 2003; Fryirs, 2013). Several studies conducted a detailed analysis of sediment connectivity while identifying sediment sources, deposits and pathways using combinations of mapping and modelling techniques (Keesstra *et al.,* 2009; Lexartza-Artza & Wainwright, 2011; Marchamalo *et al.,* 2015). In the TGRA, the demand was only partially addressed in recent attempts of soil erosion modelling (Shi *et al.,* 2012).

Empirical soil erosion models, such as the Universal Soil Loss Equation (USLE; Wischmeier & Smith, 1981) and the Revised Universal Soil Loss Equation (RUSLE; Renard *et al.,* 1997) were extensively applied in the TGRA (Shi *et al.,* 2004; Zhang, 2008; Strehmel *et al.,* 2015). The USLE/RUSLE establishes relationships between rainfall, topography, conservation practices, soil and vegetation to estimate long-term annual averages of sheet and rill erosion (Zhou *et al.,* 2008). Soil loss is calculated from the product of environmental coefficients, which were derived from field observations in 37 US states at plot scale (Zhang *et al.,* 1996; Shen *et al.,* 2009; Terranova *et al.,* 2009). The USLE/RUSLE predicts an average soil loss for an extended time period, provided that the application remains within the range of conditions for which the model was developed (Grønsten & Lundekvam, 2006). However, erosive effects of complex topography are not included, because the influence of flow convergence and divergence is not adequately regarded (Mitasova *et al.,* 1996; Tarboton, 1997; Capolongo *et al.,* 2008). Beyond, these approaches are incapable to provide estimations of spatial erosion structures, while deposition is disregarded at all (Zhang *et al.,* 1996).

A few studies applied physically based erosion and sediment transport models in the TGRA, such as the European Soil Erosion Model, the Water Erosion Prediction Project (WEPP) or the Water and Tillage Erosion Model (WaTEM; Cai *et al.,* 2005; Shen *et al.,* 2010; Shi *et al.,* 2012). These models include the spatial variability of the erosion processes and provide spatially distributed outputs of erosion and deposition. They are based on simulating the individual components of the erosion processes by solving the corresponding mass equations (Zhang *et al.,* 1996; Aksoy & Kavvas, 2005). Thus, a variety of spatially distributed input data with respect to soil conditions, terrain and land use is required. The application and performance of physically based models is primarily determined by the quality of the input data. In this context, an inadequate resolution or consistency may not represent the erosion-related heterogeneity of the study area (Jetten *et al.,* 2003; Jordan *et al.,* 2005). Thus, the models underperform if the complexity of the model is not in agreement with the data quality (Van Rompaey & Govers, 2002). Moreover, physically based models can be distinguished between event-based models to simulate sediment reallocations of single erosive events and continuous models, addressing a series of events (Nearing *et al.,* 2005). Input data requirements for continuous models are less restrictive compared with event-based models, because an event-specific parametrization is avoided. However, event-based models are preferable to investigate erosive responses in areas with few but high intensity rainfall such as the TGRA (Cai *et al.,* 2005). Recent erosion studies in the TGRA, using physically based erosion and deposition models, produced acceptable results (Cai *et al.,* 2005; Shen *et al.,* 2009, 2010; Shi *et al.,* 2012). However, most of the studies applied continuous models because of limited data availability at the catchment scale (Shen *et al.,* 2009, 2010; Shi *et al.,* 2012). By contrast, Cai *et al.* (2005) applied an event-based and physically based model at the plot scale that facilitates parametrization, but is inadequate to assess spatial organization of conservation measures in the catchment (Shi *et al.,* 2012).

Copyright © 2016 John Wiley & Sons, Ltd.

1099145x, 2017, 4, Downloaded from https://onlinelibrary.wiley.com/doi/10.1002/ldr.2503 by alexandre Wadoux - Bibliothèque de Sorbonne Université , Wiley Online Library on [22/01/2025]. See the Terms and Conditions (https://onlinelibrary.wiley.com/terms-and-conditions) on Wiley Online Library for rules of use; OA articles are governed by the applicable Creative Commons License

This study aims to provide a methodological framework that enables a detailed assessment of sediment reallocations due to erosive rainfall events in a data scarce and small mountainous catchment within the TGRA. To identify and characterize erosive events, we continuously monitored the catchment on rainfall, runoff and sediment yield in temporal resolution of 10 min for a period of 12 months. The reliability of the monitoring data was evaluated by a comparison with long-term observations. Erosive events were determined based on the cause–effect relationship between rainfall, runoff and sediment yield. We applied EROSION 3D that represents an event-based and physically based erosion and deposition model (Schmidt, 1991, 1992). Digital soil mapping (DSM) techniques were used to generate spatially distributed soil property information, therefore facilitating the model parametrization in an area of general data scarcity. Thus, the objective of the study is the spatial identification and characterization of sediment sources and deposits within the catchment.

MATERIAL AND METHODS

Study Area

The study was conducted in the Upper Badong catchment (Figure 1), located approximately 74 km upstream of the TGD in western Hubei Province (31°1 24 N, 110°20 35 E). The area covers 428.7 ha of which 72% are exposed to the north. Elevation ranges from 469 to 1,483 m with an average of 1,053 m. The average slope angle amounts to 26° and ranges between 0° and 53°. The lithology of the southern study area exhibits clayed siltstone linked to the middle Triassic formation, while dolomite and microcrystalline limestone from the lower Triassic is dominant in the north. According to the Chinese Soil Taxonomic system, dominant soil groups are purple soils in the south and yellow to yellow-brown soils in the north. Following the World Reference Base for Soil Resources (WRB, 2014), these soils refer to Cambisols and Alisols, respectively. The climate is humid subtropical with an average temperature of 12.9°C and an average annual precipitation of 1,082 mm. The rainfall regime of the study area shows a unimodal distribution with 68% of rainfall occurring from May to September. Land use is dominated by secondary forest vegetation (79.4%) in the steep hillslopes of the mountainous study area. Small agricultural plots (<0.5 ha) are scattered in the study area but concentrate to unconsolidated farmland patches in the north at elevations between 500 and 700 m, in the middle-east from 850 to 1,050 m, and in the south from 1,150 to 1,250 m. Because the climate allows two crops per year, the main patterns of crop rotation show corn and sweet potato in summer, followed by rapeseed and cabbage in winter. Conservation farming practices, such as contour tillage, furrow-ridge tillage or mulching with crop residues after harvest, are increasingly implemented.

Data Acquisition and Preparation

We established a monitoring network to continuously record data on rainfall, runoff and sediment yields (Figure 1). The data were logged in a temporal resolution of 10 min for a period of 12 months, starting in June 2013.

Rainfall data were obtained by two self-emptying tipping bucket rain gauges using the Vantage Pro 2 system by Davis Instruments (Hayward, CA , USA) with a single impulse capacity of 0.2 mm. The calibrated rain gauges were installed at elevations of 501 and 1,193 m within the study area (Figure 1). The orifices were positioned at 1.5 m above ground to avoid disturbance by vegetation and wind. For further processing, data from both rain gauges were

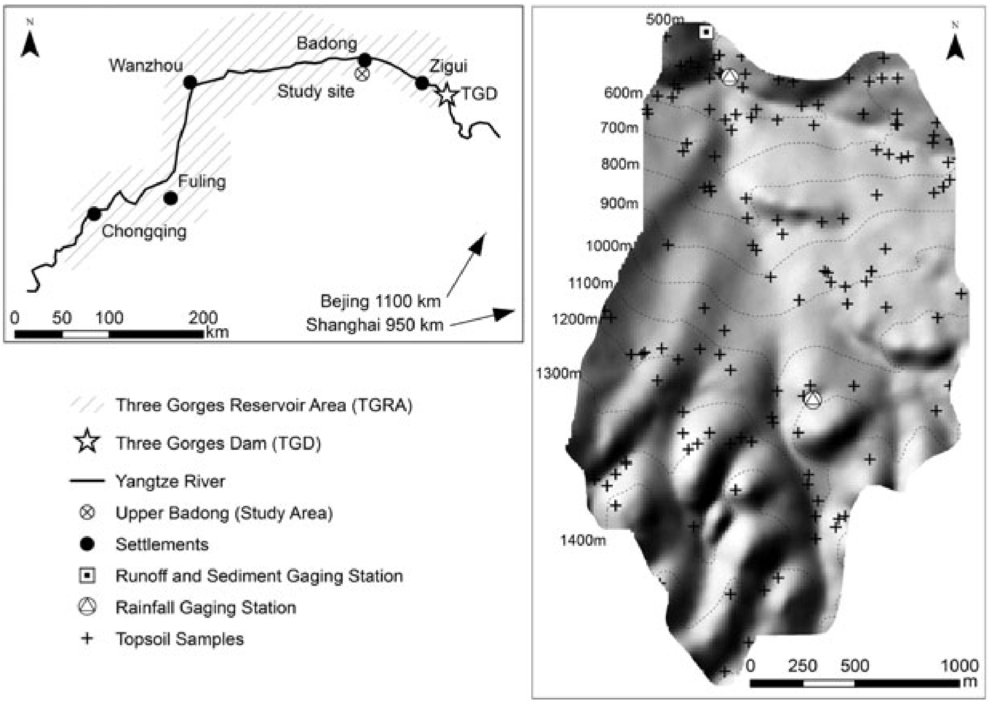

Figure 1. Study area (right) and its location within the Three Gorges Reservoir Area (left).

1099145x, 2017, 4, Downloaded from https://onlinelibrary.wiley.com/doi/10.1002/ldr.2503 by alexandre Wadoux - Bibliothèque de Sorbonne Université , Wiley Online Library on [22/01/2025]. See the Terms and Conditions (https://onlinelibrary.wiley.com/terms-and-conditions) on Wiley Online Library for rules of use; OA articles are governed by the applicable Creative Commons License

averaged. We compared the measured short-term data with long-term monthly records to evaluate their representativeness. The long-term records were obtained between 1960 and 2009 by the China Meteorological Administration (CMA, 2012) at a climate station (ID 57355) in distance of approximately 7 km from the study area. The analysis was accomplished by comparing the distributions of the data regimes using descriptive statistics.

Runoff data were obtained using a water pressure sensor (PTM/N/SDI-12 by STS-Sensors) that was positioned at the outlet of the catchment (Figure 1). Primarily, the water level was derived based on the water pressure and the geometry of the flow cross-section, which was measured in-situ. Subsequently, we applied the standard flow rate equation to determine the runoff (Kirkby, 1978):

$$Q = A{*}V_q, \qquad (1)$$

where: Q is the runoff (m^3s^{-1}), A is the flow cross-section (m^2) and V_q is the average flow velocity (ms^{-1}). V_q was calculated according to the empirical Manning equation for gravity flow in open channels (Kirkby, 1978):

$$V_q = \frac{1}{n}{*}\delta^{2/3}s^{1/2}, \qquad (2)$$

where: n is the Manning coefficient for the hydraulic surface roughness ($sm^{-1/3}$), s is the slope gradient (−) and δ is the flow depth (m).

We obtained sediment yield data from the outlet using a turbidity sensor (SN-PNEPA by PONSEL).The sensor measures the light intensity with an infrared beam that is scattered because of suspended particles. The measure is expressed in nephelometric turbidity units (NTU) and indicates the clarity of the water, which is mainly influenced by suspended sediments from eroding soil (Satterland & Adams, 1992; Anderson, 2005). Because the NTU measure depends on the properties of the suspended sediment, a conversion into mass units ($mg\,L^{-1}$) requires a site-specific calibration. Thus, we progressively added 250 composite sediment samples from the entire study area to a defined water volume of 5 L. By stepwise NTU measurements, we derived a calibration curve to convert NTU values to suspended sediment load in mass units.

During three field campaigns between 2012 and 2014, we conducted singular topsoil (0–20 cm depth) moisture measurements (TDR-sensor ML3 Thetakit by Delta-T Devices) at 235 sites randomly distributed over the entire study area. We further collected 140 topsoil samples (0–25 cm depth) according to statistical sampling designs, which adequately enable the generation of soil property maps by DSM techniques (Stumpf *et al.,* 2015a, 2015b). The samples were analyzed for organic carbon content, particle size distribution and bulk density, because these parameters outline relevant soil information for the application of EROSION 3D. At each sampling location, we pooled five sub samples from the corners and the center of a 40 × 40 cm square to obtain composite samples. In addition, we pooled three sub samples, which were randomly obtained within this square, using a cylindrical core cutter with a defined volume of 100 cm^3. We used aliquots (50 g) of the homogenized and dried (40 °C) composite samples to determine the soil organic carbon content (elemental analyzer Vario EL III). The remains of the composite samples were used for particle size analysis. The samples were sieved (<0.63 mm) to separate sand contents, while silt and clay contents were separated using the Sedigraph III 5120 by micromeritics GmbH. The bulk density was derived from the dry weight (105 °C) of the volume defined samples.

Land use information was based on a RapidEye satellite image from 28 September 2012, providing five spectral bands in a spatial resolution of 5 × 5 m (RapidEye, 2012). We derived six land use classes according to 'cropland', 'grassland', 'broadleaf', 'conifer', 'shrub', 'woods' and 'built up' (Liu *et al.,* 2005). During the field campaigns in 2013 and 2014, we further refined the land use class 'cropland' according to occurring crop rotations into 'corn-rapeseed' and 'potato-cabbage' based on *in situ* observations. Moreover, we generated a digital elevation model (DEM) based on digitizing a topographical map of the catchment with contour lines at 10 m intervals. The data were rasterized and resampled to a cell size of 25 × 25 m to buffer potential uncertainties.

Determination of Erosive Events

We used monitoring data on rainfall, runoff and sediment yield to determine rainfall–runoff events and their associated erosive response. In a first step, we disaggregated the rainfall record according to a minimum inter-event time of 6 h using the R-package 'hydromad' (Andrews & Guillaume, 2015). This threshold is commonly applied in event-based erosion studies (Wischmeier & Smith, 1981; Xie *et al.,* 2002; Bagarello *et al.,* 2008; Soulis *et al.,* 2009; Taguas *et al.,* 2011) to identify independent rainfall events with similar initial soil moisture conditions that control runoff generation (Bracken *et al.,* 2008; Todisco, 2014). In a second step, we determined the direct runoff associated with each rainfall event. Direct runoff originates from rainfall that contributes immediately to the streamflow, while baseflow reaches the streamflow with a substantial delay (Merz *et al.,* 2006). We applied a recursive digital filter technique on the runoff record to separate baseflow from direct runoff (Nathan & McMahon, 1990; Arnold *et al.,* 1995). Using the R-package 'Ecohydrology' (Fuka *et al.,* 2014), the following filter equation was applied:

$$q_t = \beta{*}q_{t-1} + \frac{1+\beta}{2}{*}(Q_t - Q_{t-1}), \qquad (3)$$

where: q_t ($m^3\,s^{-1}$) is the filtered direct runoff at the time step t (min), Q_t ($m^3\,s^{-1}$) is the original streamflow and β (−) is the filter parameter.

While an initial rainfall impulse defined the start of a rainfall–runoff event, the end was indicated when no longer associated direct runoff occurred. Using this event distinction, we attributed the respective sediment yield to each

Copyright © 2016 John Wiley & Sons, Ltd.

1099145x, 2017, 4, Downloaded from https://onlinelibrary.wiley.com/doi/10.1002/ldr.2503 by alexandre Wadoux - Bibliothèque de Sorbonne Université , Wiley Online Library on [22/01/2025]. See the Terms and Conditions (https://onlinelibrary.wiley.com/terms-and-conditions) on Wiley Online Library for rules of use; OA articles are governed by the applicable Creative Commons License

rainfall–runoff event to identify their erosive response. The adequacy of this procedure depends on the strength of the cause–effect relationship between rainfall, runoff and sediment yields within the specific catchment (Todisco *et al.*, 2014). This was evaluated by comparing the regimes of the monitoring data over the measuring period, using a correlation matrix.

For further analyses, we selected these events, which exhibit direct runoff and an associated erosive response. In contrast, we rejected those events where a distinct attribution of direct runoff to a rainfall event was impossible because of inadequate separation between direct runoff and baseflow (Blume *et al.,* 2007).

Each of the erosive rainfall–runoff events was characterized according to the event-triggering rainfall properties (Table II), such as total rainfall amount P (mm), the duration of occurring rainfall t_p (h), the maximum rainfall intensity in 30 min I_{30} (mm*h^{-1}), the maximum rainfall intensity in 60 min I_{60} (mm*h^{-1}) and the erosivity EI_{30} calculated as follows (Brown & Foster, 1987):

$$EI_{30} = \sum_{r=1}^{0} E^* I_{30}, \tag{4}$$

where: E (MJ2 ha^{-1}) is the rainfall kinetic energy for a time interval r that can be estimated by

$$E = 0.29^*[1 - 0.72^*\exp(-0.05 i_r)]^* P_r. \tag{5}$$

Modelling Sediment Reallocation

We applied EROSION 3D (Schmidt, 1991, 1992), a raster-based and physically based erosion and deposition model that calculates soil losses and deposition, initiated by single rainfall events or event sequences in small watersheds. The model includes the erosional processes of direct runoff, detachment of soil particles by rainfall splash and runoff, transport of detached particles by runoff, routing of runoff and sediment and sediment deposition. The mathematical incorporation of these processes is based on two subroutines, addressing runoff and more explicitly erosion.

The runoff subroutine calculates the rainfall excess according to a modified Green and Ampt infiltration equation (Green & Ampt, 1911; Weigert & Schmidt, 2005; Schindewolf & Schmidt, 2012):

$$i = k_s{}^* g + k_s{}^* \frac{\Psi_{m0}}{\sqrt{\frac{2k_s{}^*\Psi_{m0}{}^*t}{P_f{}^*(\Theta_s - \Theta_0)}}}, \tag{6}$$

where: i is the infiltration rate [kg/(m^2 s)], k_s is the saturated hydraulic conductivity [(kg s) m^{-3}], g is the gravity (m s^{-2}), Ψ_{m0} is the matric potential (N m kg^{-1}) related to the initial water content Θ_0 (N m kg^{-1}), t is time (s), P_f is fluid density (kg m^{-3}) and Θ_s is the saturated water content (m^3 m^{-3}). The saturated hydraulic conductivity k_s is estimated using the pedotransfer functions according to Campbell (1991):

$$k_s = 4^*10^{-3*}\left(1.3^*\frac{10^{-3}}{P_b}\right)^{1.3^*b} {}^*\exp(-0.069^*T - 0.037^*U, \tag{7}$$

with

$$b = \left(10^{-3*}D\right)^{-0.5} + 0.2^*\delta_p, \tag{8}$$

where: P_b is the bulk density (kg m^{-3}), T is the clay content (kg kg^{-1}), U is the silt content (kg kg^{-1}), b is parameter ($^-$), D is the average diameter of soil particles (m) and δ_p is the standard deviation of the average diameter of soil particles ($^-$).

Because the pedotransfer function assumes a rigid soil matrix, the temporal variability of the soil structure is disregarded. Therefore, the saturated hydraulic conductivity k_s is corrected by a multiplication with an empirically derived so-called skinfactor to adjust the infiltration rates (Schindewolf & Schmidt, 2012).

The erosion subroutine, thus the spatial quantification of particle detachment, transport and deposition, is based on the momentum-flux approach (Schmidt, 1991). Basically, this approach follows the assumption that the erosive impact of direct runoff and rainfall splash is proportional to their exerted momentum fluxes (Schindewolf & Schmidt, 2012). Where the momentum flux of the direct runoff flow φ_q is defined by

$$\phi_q = \frac{W_q{}^* V_q}{\Delta X}, \tag{9}$$

the momentum flux of the rainfall splash $\phi_{r,\alpha}$ follows the equation

$$\varphi_{r,\alpha} = W_r{}^* V_r{}^* \sin\alpha^*(1 - CL), \tag{10}$$

<u>*where:*</u> W_q and W_r are the mass rate of direct runoff flow respectively rainfall splash, V_q and V_r are the average flow velocity respectively velocity of the rainfall droplets, Δx is the length of the slope segment, α is the slope angle and CL the soil cover.

Because an observable rate of erosion requires a minimum rate of direct runoff q_{crit}, the erosion resistance of the soil is defined as the critical momentum flux φ_{crit}, following the equation (Schindewolf & Schmidt, 2012)

$$\varphi_{crit} = \frac{q_{crit}{}^* P_q{}^* V_q}{\Delta X}, \tag{11}$$

where: q_{crit} is the volume rate of flow [m^3 (ms)$^{-1}$] at initial erosion, P_q is the fluid density (kg m^{-3}), Δx is the slope segment width (m) and V_q is the flow velocity (m s^{-1}) that we derived according to Equation (2).

Besides the obtained rainfall records and the DEM, EROSION 3D requires a set of raster-based soil property maps that represent their spatial heterogeneity at catchment scale as good as possible. The parameters of particle size distributions according to textural classes (%), bulk density

Copyright © 2016 John Wiley & Sons, Ltd.

1099145x, 2017, 4, Downloaded from https://onlinelibrary.wiley.com/doi/10.1002/ldr.2503 by alexandre Wadoux - Bibliothèque de Sorbonne Université , Wiley Online Library on [22/01/2025]. See the Terms and Conditions (https://onlinelibrary.wiley.com/terms-and-conditions) on Wiley Online Library for rules of use; OA articles are governed by the applicable Creative Commons License

(kg m^{-3}) and organic carbon content (%) were assumed to be steady over the period of interest. In contrast, the parameters soil cover (%), erosion resistance (N m^{-2}), hydraulic roughness according to Manning's n (N m$^{-1/3}$), skinfactor ($^{-}$) to correct infiltration rates and the initial soil moisture (vol.−%) were adjusted for each event (Schmidt *et al.,* 1999; Schindewolf & Schmidt, 2012).

Digital soil mapping was applied to derive the steady soil parameters, because DSM techniques are cost-efficient and provide soil property maps in high resolution (McBratney *et al.,* 2003; Behrens *et al.,* 2010; Behrens *et al.,* 2014; Zhu *et al.,* 2015). Generally, DSM enables to obtain the spatial distribution of soil properties by linking soil samples at points with correlated and colocated environmental predictor covariates using classification or regression rules (McBratney *et al.,* 2003). We applied random forest (RF) regression, an ensemble classifier that is based on averaging the results of multiple randomized decision tree models for the final estimations (Peters *et al.,* 2007; Breiman, 2001). RF was selected because it includes an internal error estimation and has been successfully applied in the field of DSM (Peters *et al.,* 2007; Heung *et al.,* 2014; Stumpf *et al.,* 2015b). We set-up RF regression models for each required steady soil parameter using the 140 topsoil samples and a pool of continuous terrain covariates, because RF is robust to noise and multi-collinearity in the predictors (Díaz-Uriarte & De Andrés, 2006). The covariates were derived by a digital terrain analysis using the SAGA GIS-toolbox 'Terrain Analysis – Morphometry/Hydrology' (SAGA, 2012). For processing RF, we applied the R-package 'randomForest' (Liaw & Wiener, 2002; Table I).

The soil cover was estimated by interpreting the refined land use map in combination with crop rotation patterns and the seasonally occurring grow stages. The input parameters erosion resistance, hydraulic roughness and skinfactor were estimated using a parameter catalogue (Michael, 2000). This catalogue contains a progressively updated compilation of empirically obtained parameter values for different soils and crops, considering seasonal variations and management practices (Schmidt *et al.,* 1999; Schindewolf & Schmidt, 2012). The parameter soil moisture is most sensitive and highly variable in time and space (Schmidt, 1992; Starkloff & Stolte, 2014). Thus, we initially used the average soil moisture values from the parameter catalogue. Then, we iteratively ran the EROSION 3D model with varying soil moisture values and selected the best fit between observed and predicted direct runoff at the outlet (Jetten *et al.,* 1999; Schmidt *et al.,* 1999). Because of the average moisture values of the test model runs were only allowed to deviate by 20% from the average observed data, we ensured that the final moisture setting remained in realistic limits.

For the EROSION 3D model runs, all input parameter were harmonized to a cell size of 25 × 25 m. We validated the model output of each event individually by comparing the predicted sediment yield at the catchment outlet with the observed sediment yield. To express the discrepancy, we used the proportional deviation (ERR) of the prediction from the respective observation. Moreover, we assessed the average model performance for the sequence of erosive events using the averaged individual prediction error ($\mathrm{ERR}_{\mathrm{average}}$), the root mean square error (Willmott & Matsuura, 2005) and the Nash–Sutcliff coefficient (NS; Nash & Sutcliff, 1970; Krause *et al.,* 2005). The equations of the quality measures are defined as follows:

$$ERR = \frac{|SY_{obs} - SY_{sim}|}{\overline{SY_{obs}}}, \tag{12}$$

$$RMSE = \sqrt{\frac{\sum_{i=1}^{n}\left(SY_{sim,i} - SY_{obs,i}\right)^2}{n}}, \tag{13}$$

$$NS = 1 - \frac{\sum_{i=1}^{n}\left(SY_{obs,i} - SY_{sim,i}\right)^2}{\sum_{i=1}^{n}\left(SY_{obs,i} - SY_{ave,obs,i}\right)^2}, \tag{14}$$

where: SY_{obs} (Mg) is the observed sediment yield of an event i, SY_{sim} (Mg) is the predicted sediment yield and $SY_{ave,obs}$ (Mg) is the average observed sediment yield. For model validation, we used the R-package 'hydroGOF' by Zambrano-Bigiarini (2014).

Finally, we mapped the erosion and deposition patterns as a budget over the erosive events to spatially identify sediment sources and deposits. We interpreted the results in context of the occurring land use and topography. The model results of each pixel were separated according to four classes (severe: >50 t ha^{-1}; high: 20–50 t ha^{-1}; moderate: 10–20 t ha^{-1}; low: 0–10 t ha^{-1}) for erosion and deposition, respectively. This classification is based on thresholds that are commonly applied in the TGRA with respect to erosion studies (Shi *et al.,* 2004; Shi *et al.,* 2012).

Table I. Summary statistics (min: minimum; max: maximum; average; SD: standard deviation) and accuracy (coefficient of determination: R^2; root mean squared error: RMSE) of steady soil parameters

	Min	Max	Average	SD	RMSE	R^2
Clay content (%)	18.6	44.8	32.6	3.7	5.1	0.58
Silt content (%)	43.5	69.0	62.3	3.9	5.0	0.57
Sand content (%)	1.4	30.8	4.9	5.2	3.9	0.79
Bulk density (kg m^{-3})	700	2,000	1,200	100	100	0.36
Organic carbon (%)	1.0	3.2	2.1	0.4	0.5	0.45

Copyright © 2016 John Wiley & Sons, Ltd.

1099145x, 2017, 4, Downloaded from https://onlinelibrary.wiley.com/doi/10.1002/ldr.2503 by alexandre Wadoux - Bibliothèque de Sorbonne Université , Wiley Online Library on [22/01/2025]. See the Terms and Conditions (https://onlinelibrary.wiley.com/terms-and-conditions) on Wiley Online Library for rules of use; OA articles are governed by the applicable Creative Commons License

Table II. Properties of selected and excluded events. The duration (D), the rainfall amount (P), the peak intensity of 60-min and 30-min intervals (I_{60}, I_{30}), the erosivity (EI_{30}) and the observed sediment yield (SY_{obs}) are applied to characterize the events

		Time	D (min)	P (mm)	I_{60} (mm/60 min)	I_{30} (mm/30 min)	EI_{30} (MJ ha^{-1}*I_{30})	SY_{obs} (Mg)
Selected for further analysis	SY < 1 Mg	20.06.2013	100	13.2	12.6	11.6	88.1	0.43
		29.07.2013	220	17.2	9.2	7.8	52.2	0.92
		18.08.2013	100	6.8	6.4	6.4	23.4	0.34
		25.08.2013	340	11.2	7.4	6.8	19.9	0.88
		27.03.2014	50	9.8	9.8	8.0	26.4	0.18
		28.03.2014	200	19.8	13.0	7.6	53.8	0.79
		01.05.2014	530	14.6	7.8	5.2	8.8	0.73
		24.05.2014	310	11.8	7.0	4.4	5.9	0.62
	SY > 1 Mg	22.06.2013	170	28.6	22.2	16.4	501.7	4.21
		30.06.2013	100	14.2	13.6	13.6	154.5	1.03
		05.07.2013	530	39.4	26.0	17.6	516.5	3.88
		21.07.2013	270	19.8	14.2	12	116.9	2.58
		03.08.2013	400	22.0	21.6	15.2	286	1.19
		28.08.2013	720	42.6	34.8	32.4	2789.9	7.03
Excluded		05.06.2013	1,158	49.2	13.6	9.6	120.2	—
		24.06.2013	1,458	36.4	7.0	4.6	22.5	—
		23.08.2013	2,368	25.0	8.0	5.6	17.0	—
		09.09.2013	1,692	41.2	9.0	7.4	48.4	—
		20.04.2014	1,422	42.0	13.8	9.4	81.1	—

RESULTS

Evaluation of the Monitoring Data

We compared the monitored rainfall data (June 213 to May 2014) to the long-term records from Badong climate station (January 1960 to December 2009; Figure 2). With 895 mm, the total rainfall amount of the short-term records is less compared with the long-term annual average of 1,082 mm, revealing relatively dry conditions during the monitoring period. This decline is attributable to the months of May, July and October, showing a reduced rainfall amount between 54 and 68 mm. The deviations of the remaining months range between 1 mm in January and 20 mm in June with a positive budget of 11 mm. Furthermore, we compared

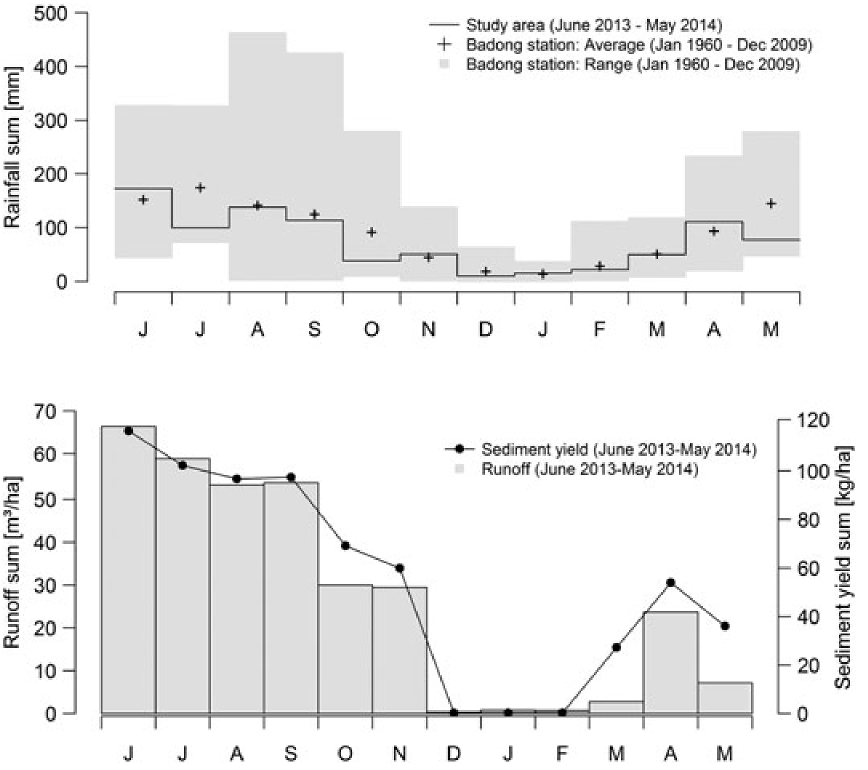

Figure 2. Regimes of monthly rainfall, runoff and sediment yield data in the monitoring period. Rainfall records are compared with ranges and averages of long-term records from Badong station (above). Regimes of runoff and sediment yield are compared among each other (below).

Copyright © 2016 John Wiley & Sons, Ltd.

1099145x, 2017, 4, Downloaded from https://onlinelibrary.wiley.com/doi/10.1002/ldr.2503 by alexandre Wadoux - Bibliothèque de Sorbonne Université , Wiley Online Library on [22/01/2025]. See the Terms and Conditions (https://onlinelibrary.wiley.com/terms-and-conditions) on Wiley Online Library for rules of use; OA articles are governed by the applicable Creative Commons License

the short-term amounts per month with the long-term average maxima and minima per month. The results show that the obtained records were all within the range of the long-term records (Figure 2). Both short-term and long-term records exhibit a unimodal distribution with 67% and 68% of the annual rainfall amounts occurring during the wet season from May to September. The analogy between the rainfall records reveal that the short-term record is representative for the area.

Moreover, we evaluated the interrelation between the monitored rainfall, runoff and sediment records within the study area (Figure 2). The annual distribution of the obtained runoff sums and sediment yields per month corresponds to the recorded rainfall regime. With a total annual runoff of 324 $m^3 ha^{-1}$, 80% occurs in the wet season from May to September. We found the maximum runoff in June with 66 $m^3 ha^{-1}$, while from December to January, less than 1 $m^3 ha^{-1}$ runoff was recorded. Similarly, the total annual sediment yield adds up to 666 kg ha^{-1}, of which 71% occurs in the wet season. June shows the maximum yield with 116 kg ha^{-1}, while minima occur from December to February with less than 3 kg ha^{-1} (Figure 2). We further calculated the relation of rainfall, runoff and sediment yield by using the correlation coefficient. With a resolution of 10 min, rainfall data exhibit an r of 0.94 to runoff and 0.89 to sediment yield data, while the latter two are correlated with $r = 0.84$. The similar regimes of the recorded data and the associated r-values (>0.80) approve a strong cause–effect relationship between rainfall, runoff and sediment yield.

Properties of Erosive Rainfall Events

During the monitoring period, we identified 19 erosive events, of which we excluded five from further analyses (Table II). Compared with the selected events and referring to the average properties, the excluded events show an increase in duration D and total rainfall amount P of 1,140 min and 19.4 mm, respectively. The average peak intensities I_{60}, I_{30} are lower by 4.6 mm 60 min^{-1} and 4.5 mm 30 min^{-1}, respectively, and the average erosivity EI_{30} are lower by 273.9 MJ ha^{-1} mm h^{-1}. Moreover, the excluded events show intra-event periods of no rainfall close to the inter-event time of 6 h. Summarizing, the excluded events generally exhibit increased durations with decreased intensities of discontinuous rainfall. These patterns result in temporally variable runoff generation, thus, preventing an adequate separation between direct runoff and baseflow.

The average properties of the 14 selected events show a duration D of 276 min, a total rainfall of 19.4 mm, peak intensities I_{60} and I_{30} of 14.7 and 11.8, and an erosivity EI_{30} of 331.7 MJ ha^{-1} mm h^{1}. These event properties resulted in sediment yields SY_{obs} ranging between 0.18 and 7.03 Mg with an average of 1.77 Mg and a total sediment yield of 24.8 Mg. Eight events show a sediment yield less than 1 Mg with an average of 0.61 Mg (small events). Six events show sediment yields above 1 Mg with an average of 3.32 Mg (large events; Table II). The large events account for 80.3% of the sediment yield and 61% of the total rainfall amounts over all events.

Model Performance

To evaluate the model performance for each event, we derived the individual model error (ERR). We compared the ERR values to the observed sediment yields (Figure 3). Generally, small events with SY below 1 Mg exhibit increased model errors compared with events with sediment yields above 1 Mg. The average model error ($ERR_{average}$) of the eight small events amounts to 36.2%, ranging between 15.1% and 62%. Except for one event, the ERR refer to severe underprediction. In contrast, the six large events show an average model error of 7.5%, ranging from 0.1% to 14.7%. Those values mainly result from overprediction. The simulated *SY* for all events amounts 24.2 Mg and deviates by 2.3% from the observed sediment yield of 24.8 Mg. The $ERR_{average}$ of all events results in 23.9%. Comparing the observed versus the predicted sediment yields across all events show a NS-value of 0.98 and a root mean square error of 0.27 (Figure 3). Thus, the average model quality is high. However, the evaluation of the individual model errors reveals ambiguous patterns. While the model results for large events (>1 Mg) show low ERR errors, the model runs for the small events (<1 Mg) result in increased ERR errors.

Characteristics of Sediment Reallocations

We mapped the model results according to four classes of 'severe' (>50 Mg ha^{-1}), 'high' (20–50 Mg ha^{-1}), 'moderate' (10–20 Mg ha^{-1}) and 'low' (<10 Mg ha^{-1}) erosion and deposition, respectively (Figure 4). Moreover, we evaluated the spatial extent of erosion and deposition zones (Table III) according to land use patterns (Table IV). Because the large events with a SY > Mg account for the vast majority of all events (80.3%) and uncertainties of the small events (<1 Mg) are high ($ERR_{average} = 36.2\%$), we excluded the latter from the spatial analysis.

Agricultural land (land use classes: 'corn-rapeseed', 'potato-cabbage' and 'grassland') presents 17% (73.2 ha) of the total study area (428.7 ha). In 79.4% (340.4 ha) of the area 'mixed forest', composed of the land use classes 'broadleaf', 'conifer', 'woods' and 'shrub' occurs. A small proportion of 3.5% (15.1 ha) is dedicated to small farm buildings.

Corresponding to the largest coherent agricultural area, 'severe' and 'high' erosion is predominantly located in the lowlands of the north, where the elevation ranges between 500 and 650 m. A further band of 'severe' and 'high' erosion extends south of the major agricultural area at elevations between 800 and 900 m. 'Low' erosion mainly occurs on the agricultural areas in the south at elevations between 900 and 1,400 m. Average slope inclinations of the erosion area amount to 21.5°, ranging between 4.3° and 39.4°. The mapping shows erosion on an area of 47.5 ha, accounting for 11.1% of the entire study area (Figure 4). The simulated erosion rate ranges between 0.01 and 527 Mg ha^{-1} with an average of 49.9 Mg ha^{-1} referring to the erosion area. A

Copyright © 2016 John Wiley & Sons, Ltd.

1099145x, 2017, 4, Downloaded from https://onlinelibrary.wiley.com/doi/10.1002/ldr.2503 by alexandre Wadoux - Bibliothèque de Sorbonne Université , Wiley Online Library on [22/01/2025]. See the Terms and Conditions (https://onlinelibrary.wiley.com/terms-and-conditions) on Wiley Online Library for rules of use; OA articles are governed by the applicable Creative Commons License

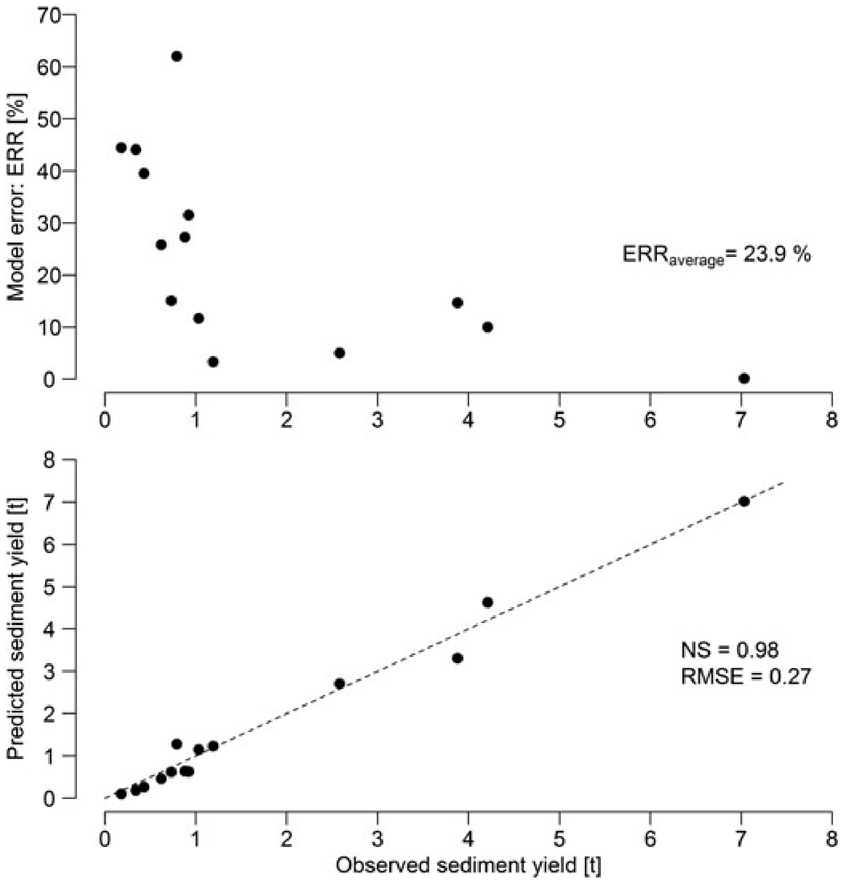

Figure 3. Individual and average model performance for all erosive events (black dots). Performance variability is expressed by comparing the individual prediction error (ERR) to the observed sediment yields (above). Average model performance is revealed by the averaged individual prediction error (ERR$_{average}$, above) and by comparing observed to predicted sediment yields, applying the Nash–Sutcliffe coefficient (NS) and the root mean square error (RMSE; below).

proportion of 37.5% (17.8 ha) of the erosion area is classified as 'severe' and 45.1% (21.4 ha) as 'low' (Table III). In terms of land use, erosion mainly occurs on 'corn-rapeseed' and 'potato-cabbage' with approximately 69% of the respective area (Table IV).

'Severe' and 'high' sediment depositions mainly occur in the two major erosive areas in the north. Depositions are concentrated lateral of topographical depression channels, at field borders with high vegetation cover, and in infrastructural areas. In the south of the study area, at an elevation between 900 and 1,400 m, depositions are located adjacent to erosive areas and mainly classified as 'low'. The total deposition area outlines an average slope inclination of 19.8°, ranging from 1.1° to 35.5°, therefore showing a marginal decline compared with the erosion area. Deposition occurs on 47.3 ha, thus 11% of the study area (Figure 4). The deposition rate ranges between 0.01 and 499.5 Mg ha^{-1} with an average of 40.3 Mg ha^{-1}. A proportion of 61.5% (29.1 ha) of the deposition area is classified as 'low', while the remaining classes occupy a real proportions between 9.3% (4.4 ha) and 15.8% (7.5 ha; Table III). Referring to land use, deposition occurs on each class, while 'built' is occupied by 41.7% (6.3 ha) of the area. The proportional deposition area of the remaining land use classes ranges between 9.7% (33.0 ha) for 'mixed forest' and 18.4% (1.1 ha) for 'grassland' (Table IV).

DISCUSSION

Erosive Events and Data Quality

In this study, rainfall–runoff events were delimited by an initial rainfall impulse and the remission of the associated direct runoff. This procedure is in accordance with other studies on rainfall–runoff events (Baltas *et al.,* 2007; Blume *et al.,* 2007). Subsequently, observed sediment yields were attributed to identify the respective erosive response. However, the event properties that determine the erosive response highly depend on methodological settings for data acquisition, event exclusion, event classification rules and the inter-event time to disaggregate a rainfall record (Dunkerley, 2008). Todisco *et al.* (2014) argued that the event properties change in time and space and can be referred to as arbitrary because of customized settings for individual applications and environments. This limits the comparability in terms of event-based erosion studies.

In addition, high-quality monitoring data on erosion become increasingly important to enable an evaluation of the site-specific cause–effect relationship and to address the requirements of complex model structures (Aksoy & Kavvas, 2005; Fang *et al.,* 2013). Commonly, continuous and high-resolution data on rainfall and runoff are available. Contrary, continuous data on sediment loads are often

Copyright © 2016 John Wiley & Sons, Ltd.

1099145x, 2017, 4, Downloaded from https://onlinelibrary.wiley.com/doi/10.1002/ldr.2503 by alexandre Wadoux - Bibliothèque de Sorbonne Université , Wiley Online Library on [22/01/2025]. See the Terms and Conditions (https://onlinelibrary.wiley.com/terms-and-conditions) on Wiley Online Library for rules of use; OA articles are governed by the applicable Creative Commons License

1099145x, 2017, 4, Downloaded from https://onlinelibrary.wiley.com/doi/10.1002/ldr.2503 by alexandre Wadoux - Bibliothèque de Sorbonne Université , Wiley Online Library on [22/01/2025]. See the Terms and Conditions (https://onlinelibrary.wiley.com/terms-and-conditions) on Wiley Online Library for rules of use; OA articles are governed by the applicable Creative Commons License

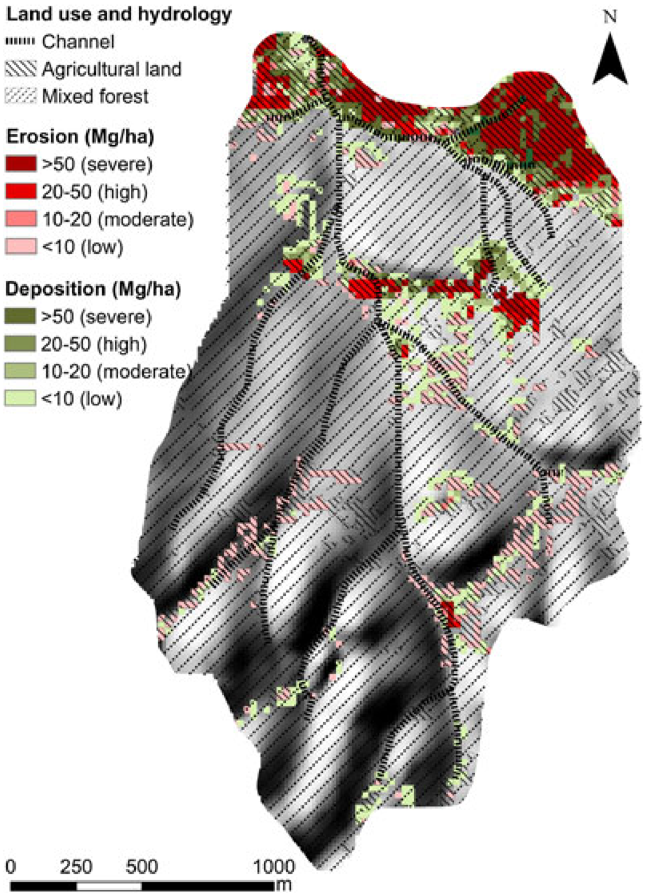

Figure 4. Sediment sources and deposits as budget across erosive events with sediment yields higher than 1 t. Depression channels and land use classes according to agricultural land (composed of the classes ‘cropland’ and ‘grassland’) and mixed forest (composed of ‘broadleaf’, ‘conifer’, ‘woods’ and ‘shrub’) are indicated. [Colour figure can be viewed at wileyonlinelibrary.com]

difficult to obtain because of required maintenance and operating personnel (Rickemann & McArdell, 2007).

Within the TGRA, Fang *et al.* (2013) investigated erosive events in a small catchment of 1,670 ha. Rainfall and runoff data were continuously measured in a resolution of 15 min. Data on sediment loads were manually obtained only during rainfall events. From a total of 205 rainfall–runoff events between 1989 and 2004, ten were classified as extreme according to a qualitative assessment of surface damage because of erosion. These events caused 83.3% of the sediment load. This supports Cai *et al.* (2005), who stated that most erosion in the TGRA is associated with very few heavy rainfall events each year.

In contrast, we continuously derived data on rainfall, runoff and sediment yield in a 10 min resolution over a period of 12 months within a catchment of 429 ha (Figure 1). The rainfall data were approved to be representative for the region, because the amounts per months were within the range of averaged daily long-term records (Figure 2). Similar to Fang *et al.* (2013), a few large events caused the major proportion of the total sediment yield. We identified 14 events of which six showed sediment yields above 1 Mg, accounting for 80.3% of the total sediment yield. Moreover, we detected high correlations between the regimes of rainfall, runoff and sediment yields with r-values above 0.8, while all regimes outline peak values during the wet season and minimum values in winter. Both the high correlations and similar distributions indicate a strong cause–effect relationship between the monitoring data.

Table III. Area of erosion and deposition (ha) and the erosion rate according to classes (severe: $>50\,\mathrm{Mg\,ha^{-1}}$, high: $20–50\,\mathrm{Mg\,ha^{-1}}$, moderate: $10–20\,\mathrm{Mg\,ha^{-1}}$, low: $<10\,\mathrm{Mg\,ha^{-1}}$)

	Total (ha)	Severe (%)	High (%)	Moderate (%)	Low (%)
Erosion	47.5	37.5	13.8	3.6	45.1
Deposition	47.3	15.8	13.4	9.3	61.5

Erosion Modelling in the Three Gorges Reservoir Area

The performance of physically based erosion models depends on the model capability to deal with the natural complexity of the erosion process and the spatial heterogeneity of the study area (De Vente & Poesen, 2005). Furthermore, the availability and quality of the input data need to be in agreement with the complexity of the model routines (Van Rompaey & Govers, 2002; De Vente *et al.,* 2013). Thus, the combined criteria of the model design, environmental conditions and data infrastructure determine the adequacy of a model for a specific research question (Boardman, 2006). In the TGRA, a few physically based model attempts have been conducted to test model performances and to assess erosion control measures at catchment scale (Shen *et al.,* 2010; Shi *et al.,* 2012).

Shi *et al.* (2012) applied WaTEM/SEDEM in catchment of 1,670 ha. This model uses the empirical RUSLE to calculate annual water erosion on hillslopes and a sediment routing along the runoff channels by incorporating local sediment transport capacity (Van Rompaey *et al.,* 2001). Shen *et al.* (2010) applied the WEPP model in a catchment of 162 ha. WEPP uses the Green-Ampt infiltration approach to simulate runoff and a steady-state sub-routine to solve a sediment continuity equation at a peak runoff rate (Flanagan & Nearing, 2000). We applied EROSION 3D in a catchment of 429 ha, a model that also uses the Green-Ampt infiltration equation for the runoff routine. However, the erosion routine is based on the momentum flux approach that relates the erosive impact of runoff and rainfall to their exerted momentum flux (Schmidt *et al.,* 1999; Schindewolf & Schmidt, 2012).

Both WaTEM/SEDEM and WEPP present continuous model approaches producing average values for erosion and deposition. In contrast, EROSION 3D is an event-based model, and therefore capable to assess the variability of erosive responses due to singular rainfall–runoff events. Event-based models require event-specific parametrization because of a high sensitivity to initial conditions. Thus, requirements for data quality in terms of accuracy and continuity are higher (Jetten *et al.,* 2003; Aksoy & Kavvas, 2005; Boardman, 2006). This especially accounts for the parameters that control infiltration, such as soil moisture and hydraulic

Copyright © 2016 John Wiley & Sons, Ltd.

Table IV. Erosion (Ero.) and deposition (Dep.) rates according classes (severe: >50 Mg ha^{-1}, high: 20–50 Mg ha^{-1}, moderate: 10–20 Mg ha^{-1}, low: <10 Mg ha^{-1}) for different land uses (ha)

Land use	Area (ha)	Ero. (Dep.) (%)	Ero. (Dep.) severe (%)	Ero. (Dep.) high (%)	Ero. (Dep.) moderate (%)	Ero. (Dep.) low (%)
Corn-rapeseed	47.8	69.2 (13.6)	26.1 (1.3)	10.3 (3.6)	2.3 (3.2)	30.5 (5.5)
Potato-cabbage	19.4	69.1 (11.3)	29.9 (0.5)	8.8 (4.6)	3.1 (2.1)	27.3 (4.1)
Grassland	6.0	5.0 (18.4)	−(8.3)	−(1.7)	−(1.7)	5.0 (6.7)
Mixed forest	340.4	0.5 (9.7)	−(1.4)	−(0.8)	−(0.6)	0.5 (6.9)
Built	15.1	- (41.7)	−(10.6)	−(6.6)	−(4.0)	−(20.5)

conductivity (Schmidt *et al.,* 1999; Jetten *et al.,* 2003; Shen *et al.,* 2010). Particularly in the TGRA, where major sediment reallocations are due to very few extreme events, the event-based assessment is of substantial interest in context of implementing erosion control measures to prevent hazardous impacts (Cai *et al.,* 2005; Fang *et al.,* 2013).

Continuous runoff data and discontinuous sediment yield data from the outlet were available for both model attempts by Shen *et al.* (2010) and Shi *et al.* (2012). The data was used to calibrate the WEPP model, while WaTEM/SEDEM was parametrized by available RUSLE data. Both studies were validated by the outlet data. In the present study, we used continuous data on runoff and sediment yield, therefore providing an increased data consistency. Model calibration was enabled by using DSM techniques to calculate high-resolution soil property information. Thus, DSM provided a solution to enable the parametrization of a physically and event-based erosion and deposition model at catchment scale in a generally data scarce environment (Stumpf *et al.,* 2015a). Further model parameters were based on land use data derived from satellite images and an empirically compiled parameter catalogue (Michael, 2000). Only the sensitive soil moisture parameter was adjusted using observed and predicted runoff data, a procedure that is commonly applied in event-based erosion modelling (Schmidt *et al.,* 1999; Jetten *et al.,* 2003). Similar to the WaTEM/SEDEM and WEPP approach in the TGRA (Shen *et al.,* 2010; Shi *et al.,* 2012), model performance of the presented approach was assessed using outlet data on sediment yield. However, because of the variability in the sediment delivery ratio with changing temporal and spatial scale, sediment yield data have been criticized for field erosion measurements (Boardman, 2006). Nevertheless, the assumption of a stable and high sediment delivery ratio is reasonable, because the cause–effect relationship between the monitoring data (rainfall, runoff, sediment yield) is strong ($r > 0.8$), and erosive events are mainly due to high intensity rainfall events that are reported to cause high sediment connectivity (Lexartza-Artza & Wainwright, 2011; Baartman *et al.,* 2013; Todisco, 2014; Marchamalo *et al.,* 2015).

Both physically based model attempts in the TGRA (Shen *et al.,* 2010; Shi *et al.,* 2012) showed acceptable average model accuracies with $NS = 0.65$ for the WaTEM/SEDEM approach and $NS = 0.84$ (average deviation: 3.9%) for the WEPP modelling. Contrary, the presented study, using EROSION 3D, exhibited an increased average accuracy of $NS = 0.98$ and a decreased average deviation of 2.3 over all modelled events. Moreover, because EROSION 3D is event-based, we also derived event-specific accuracies. We detected ambiguous model performances between small events (<1 Mg; $ERR_{average} = 36.2\%$) and large events (>1 Mg; $ERR_{average} = 7.5\%$).The increasing model performance for large events is in agreement with other event-based model attempts (Zhang *et al.,* 1996; Nearing *et al.,* 1999; Nearing, 2000; Gumiere *et al.,* 2011; Lee *et al.,* 2013). In this context, Jetten *et al.* (2003) argued that small-scale events are generally difficult to simulate because the deterministic character of erosion models is incapable to deal with the random component of measured data. Boardman (2006) relates the low accuracy of small-scale events to oversimplified runoff routines, which solely simulate runoff by infiltration excess and thereby underrating the erosive power of low intensity rainfall on saturated soil. Moreover, the decreased sediment connectivity of low intensity rainfall could cause variability in the outlet data, and therefore leading to biased estimation (Marchamalo *et al.,* 2015).

For the WaTEM/SEDEM approach, 'severe' and 'high' erosion occurred on 10.5% of the study area with an average erosion rate of 13 Mg ha^{-1}. Deposition was detected on 20.5%, while no classification on magnitude was conducted (Shi *et al.,* 2012). For the WEPP approach, the average erosion rate ranged between 2 and 38 Mg ha^{-1}, while quantitative information on proportional areas of erosion and deposition was not provided. In the presented study, 'severe' and 'high' erosion was found on 5.7% of the study area, while 11% were occupied by deposition. The average erosion rate is 49.9 Mg ha^{-1}, calculated over the erosion area and using the budget of the modelled events that account for 80.3% of the total sediment yield (Table V).

Other model-based erosion studies within the TGRA applied the empirical RUSLE, the semi-empirical SWAT model or radionuclide inventories of (C^{137}) in various scales (Quine *et al.,* 1999; Lu & Higgit, 2000; Shi *et al.,* 2004; He *et al.,* 2007; Zhang, 2008; Strehmel *et al.,* 2015). The estimated erosion rates ranged between 26 Mg ha^{-1} a^{-1} and 76 Mg ha^{-1} a^{-1}. Because the average erosion rate of the presented study lies within the range of other erosion studies in this region, the applied approach can therefore be considered as reliable. However, the comparability of erosion rates is limited because of scale-dependency in terms of space and time, a variety of measurement methods and the complex relationship between environmental factors and erosion

Copyright © 2016 John Wiley & Sons, Ltd.

1099145x, 2017, 4, Downloaded from https://onlinelibrary.wiley.com/doi/10.1002/ldr.2503 by alexandre Wadoux - Bibliothèque de Sorbonne Université , Wiley Online Library on [22/01/2025]. See the Terms and Conditions (https://onlinelibrary.wiley.com/terms-and-conditions) on Wiley Online Library for rules of use; OA articles are governed by the applicable Creative Commons License

Table V. Model-based erosion studies in the Three Gorges Reservoir Area, based on physical (EROSION 3D, WaTEM/SEDEM, WEPP), semi-empirical (SWAT), empirical (USLE/RUSLE) and radionuclide inventory (C^{137}) methods

Method	Area (ha)	Average erosion rate ($Mg\,ha^{-1}\,a^{-1}$)	Reference
EROSION 3D	429	49.9	presented study
WaTEM/SEDEM	1,670	13.2	Shi *et al.,* 2012
WEPP	162	2–38	Shen *et al.,* 2010
SWAT	162	27.0	Shen *et al.,* 2009
USLE	$2.3*10^6$	32.8	Zhang, 2008
RUSLE	$3.2*10^5$	52–76	Strehmel *et al.,* 2015
RUSLE	162	26–52	Shi *et al.,* 2004
C^{137}	70	45.0	Lu & Higgitt, 2000
C^{137}	0.21	51.5	Quine *et al.,* 1999
C^{137}	$1.1*10^8$	24.2	He *et al.,* 2007

(Boardman, 2006; De Vente *et al.,* 2007; Cantón *et al.,* 2011; Vanmaercke *et al.,* 2011; García-Ruiz *et al.,* 2015). In this context, García-Ruiz *et al.* (2015) compiled a data base of erosion rate studies from more than 4,000 sites worldwide and analyzed the data on their relation to (non-) environmental factors. The meta-analysis revealed general trends of positive relations to factors such as slope, annual precipitation and land use. However, the results showed high variability because the included studies comprised various spatial scales, durations of the experiments and methods. Moreover, García-Ruiz *et al.* (2015) argued that insufficient descriptions of study areas, methods and results further exacerbate the comparability of erosion studies.

Sediment Reallocation and Landuse

This study investigates rainfall-triggered sediment reallocations to enable sustainable land management at catchment scale. However, the sedimentological response of a landscape is complex, because it depends on a variety of interacting physical processes, which are related to topography, climate, soil and vegetation among others (Martínez-Mena *et al.,* 1998; Cammeraat, 2004; Puigdefábregas, 2005; Bracken & Croke, 2007; Bautista *et al.,* 2007; Bochet, 2015; Certini *et al.,* 2015; Marchamalo *et al.,* 2015).

The quantification of the landscape response is increasingly achieved using the concept of landscape connectivity, which describes the water-mediated sediment fluxes within a catchment (Bracken & Croke, 2007; Lexartza-Artza & Wainwright, 2011; Fryirs, 2013). López-Vicente *et al.* (2015) coupled the modified RMMF soil erosion model (López-Vicente & Navas, 2010) with the IC model of sediment connectivity (Borselli *et al.,* 2008) to map potential sediment reallocations. Marchamalo *et al.* (2015) presented a method to identify hotspots of sediment sources, deposits and their linkages by repeatedly field mapping after rainfall events. Keesstra *et al.* (2009) combined field surveys, site-specific expert knowledge and a sediment delivery model to establish a detailed sediment budget. However, the aforementioned approaches are accompanied by extensive field work, because detailed data on landscape features related to connectivity are difficult to derive from DEMs and remote sensing images (Marchamalo *et al.,* 2015; Lesschen *et al.,* 2009).

In contrast, the presented approach outlines a modelling framework of automated field monitoring and DSM techniques to calibrate a physically and event-based soil erosion model. The framework reduces efforts for field work and is applicable in data scarce and highly dynamic environments. However, the validation by outlet data only addresses overall model accuracy, while an uncertainty assessment of the modelled sediment reallocations is limited (Jetten *et al.,* 1999; Boardman, 2006).

Nevertheless, the spatial results of the presented modelling approach are in agreement with data on average soil loss rates of main land use types in southern China (Hill & Peart, 1998; Huang *et al.,* 1998; Xiang *et al.,* 2001; Gao *et al.,* 2004; Zheng & Zhang, 2006; Guo *et al.,* 2015). The data are based on plot-scale studies, from which an average soil loss rate of $0.38\,Mg\,ha^{-1}\,a^{-1}$ for the land use type ‘forest’, $5.5\,Mg\,ha^{-1}\,a^{-1}$ for ‘grassland’ and $35.4\,t\,ha^{-1}\,a^{-1}$ for ‘cropland’ were identified. According to a review of Hill & Peart (1998), average soil loss in southern China amounts to $0.1\,Mg\,ha^{-1}\,a^{-1}$ for ‘forest’, $2.4\,Mg\,ha^{-1}\,a^{-1}$ for ‘grassland’ and $62.4\,Mg\,ha^{-1}\,a^{-1}$ for ‘cropland’. In the present study, we found erosion on the land use classes ‘corn-rapeseed’ and ‘potato-cabbage’, while both land use classes were classified as ‘severe’($>50\,Mg\,ha^{-1}$) for approximately one third of the specific land use area (Table IV).

In addition, Takken *et al.* (1999) mapped erosion patterns and calculated the erosion rate after an extreme rainfall event for different land use types in a small catchment in Belgium. The results confirm the aforementioned erosion rates with no erosion for ‘forest’, $0.2\,Mg\,ha^{-1}$ for ‘grassland’, $53.0\,Mg\,ha^{-1}$ for ‘potato’ and $76.0\,Mg\,ha^{-1}$ for ‘corn’. Moreover, Takken *et al.* (1999) found deposition on 3.5% of the study area, while major deposition zones were concentrated along the topographical depression lines, at field borders with high vegetation, and on roads. These results are generally confirmed by the present study, while deposition occurred on each land use class, but concentrated in topographic depression lines and on infrastructure (Figure 4; Table IV).

The modelled erosion patterns of the present study demarcate areas of ‘severe’ soil loss in the north and smaller patches in the central and southern part of the catchment. These areas display the major sediment sources and indicate increased sedimentological connectivity

Copyright © 2016 John Wiley & Sons, Ltd.

1099145x, 2017, 4, Downloaded from https://onlinelibrary.wiley.com/doi/10.1002/ldr.2503 by alexandre Wadoux - Bibliothèque de Sorbonne Université , Wiley Online Library on [22/01/2025]. See the Terms and Conditions (https://onlinelibrary.wiley.com/terms-and-conditions) on Wiley Online Library for rules of use; OA articles are governed by the applicable Creative Commons License

(Verstraeten *et al.,* 2006; Marchamalo *et al.,* 2015). The results support the implementation of erosion control measures to mitigate soil degradation and hazardous effects for human livelihood in the TGRA (Xu *et al.,* 2013; Ferreira *et al.,* 2015). In this context, López-Vicente *et al.* (2013) suggests to stabilize surfaces and reduce sediment connectivity by establishing cropland terraces to retain eroded sediment and an increasing vegetation cover, particularly in periods of high intensity rainfall.

CONCLUSIONS

In this study, we analyzed sediment reallocations because of erosive rainfall events in a data scarce and small catchment of the TGRA in China. Thus, we set up a methodological workflow to parametrize EROSION 3D as a modelling tool to spatially identify sediment sources and deposits. Model parametrization was accomplished by using DSM techniques, land use maps based on satellite data and a parameter catalogue. Calibration data on rainfall, runoff and sediment yields were representative for the region and showed a strong cause–effect relationship. The majority of the total sediment yield was attributed to only six large erosive events. Erosion 3D performed well for large events, while small events showed high uncertainties. We detected high erosion rates on cropland, particularly on crop rotation areas of 'corn-rapeseed' and 'potato-cabbage'. The major areal proportions of deposition were attributed to the land use classes 'built' and 'grassland'. In summary, we presented an efficient methodological outline to meet the complex data requirements of a physically and event-based erosion model. Considering that the major sediment yields in the region are associated to a few large events, EROSION 3D can be recommended to identify sediment reallocations and relevant sites for erosion control measures in small catchments.

ACKNOWLEDGEMENT(S)

We thankfully acknowledge funding from the German Ministry of Education and Research (BMBF, grant No. 03G 0827A) for the German-Sino research collaboration YANGTZE GEO. We thank Bi Renneng from the University of Erlangen-Nuremberg, Giovanni Buzzo from the University of Trier, and Alexander Strehmel from the Christian-Albrechts-University of Kiel for data sharing. We thank all students from the China University of Geosciences (CUG) in Wuhan that were involved in field campaigns and laboratory analyses. We are also very grateful for the general support of the whole YANGTZE GEO research group.

REFERENCES

Aksoy H, Kavvas ML. 2005. A review of hillslope and watershed scale erosion and sediment transport models. *Catena* **64**: 247–271. DOI:10.1016/j.catena.2005.08.008.

Anderson CW. 2005. Turbidity: U.S. Geological survey (USGS) techniques of water-resources investigations. [http://pubs.water.usgs.gov/twri9A; accessed: September 2014]

Andrews F, Guillaume J. 2015. 'hydromad': hydrological model assessment and development. R package version 0.9-22. [http://hydromad.catchment.org/; accessed: May 2014]

Arnold JG, Allen PM, Muttiah R, Bernhardt G. 1995. Automated base flow separation and recession analysis techniques. *Ground Water* **33**: 1010–1018. DOI:10.1111/j.1745-6584.1995.tb00046.x.

Baartman JEM, Masselink R, Keesstra SD, Temme AJAM. 2013. Linking landscape morphological complexity and sediment connectivity. *Earth Surface Processes and Landforms* **38**: 1457–1471. DOI:10.1002/esp.3434.

Bagarello V, Di Piazza GV, Ferro V, Giordano G. 2008. Predicting unit plot soil loss in Sicily, south Italy. *Hydrological Processes* **22**: 586–595. DOI:10.1002/hyp.6621.

Baltas EA, Dervos NA, Mimikou MA. 2007. Technical note: determination of the SCS initial abstraction ratio in an experimental watershed in Greece. *Hydrological Earth System Sciences* **11**: 1825–1829. DOI:10.5194/hess-11-1825-2007.

Bautista S, Mayor AG, Bourakhouadar J, Bellot J. 2007. Plant spatial pattern predicts hillslope runoff and erosion in a semiarid Mediterranean landscape. *Ecosystems* **10**: 987–998. DOI:10.1007/s10021-007-9074-3.

Behrens T, Zhu AX, Schmidt K, Scholten T. 2010. Multi-scale digital terrain analysis and feature selection for digital soil mapping. *Geoderma* **155**: 175–185. DOI:10.1016/j.geoderma.2009.07.010.

Behrens T, Schmidt K, Ramirez-Lopez L, Gallant J, Zhu AX, Scholten T. 2014. Hyper-scale digital soil mapping and soil formation analysis. *Geoderma* **213**: 578–588. DOI:10.1016/j.geoderma.2013.07.031.

Berendse F, Van Ruijven J, Jongejans E, Keesstra S. 2015. Loss of plant species diversity reduces soil erosion resistance. *Ecosystems* **18**: 881–888. DOI:10.1007/s10021-015-9869-6.

Blume T, Zehe E, Bronstert A. 2007. Rainfall-runoff response, event-based runoff coefficients and hydrograph separation. *Hydrological Sciences Journal* **52**: 843–862. DOI:10.1623/hysj.52.5.843.

Boardman J. 2006. Soil erosion science: reflections on the limitations of current approaches. *Catena* **68**: 73–86. DOI:10.1016/j.catena.2006.03.007.

Bochet E. 2015. The fate of seeds in the soil: a review of the influence of overland flow on seed removal and its consequences for the vegetation of arid and semiarid patchy ecosystems. *The Soil* **1**: 131–146. DOI:10.5194/soil-1-131-2015.

Borselli L, Cassi P, Torri D. 2008. Prolegomena to sediment and flow connectivity in the landscape: a GIS and field numerical assessment. *Catena* **75**: 268–277. DOI:10.1016/j.catena.2008.07.006.

Bracken LJ, Cox NJ, Shannon J. 2008. The relationship between rainfall inputs and flood generation in south–east Spain. *Hydrological Processes* **22**: 683–696. DOI:10.1002/hyp.6641.

Bracken LJ, Croke J. 2007. The concept of hydrological connectivity and its contribution for understanding sediment transfer at multiple scales. *Earth Surface Processes and Landforms* **21**: 1749–1763. DOI:10.1002/hyp.6313.

Breiman L. 2001. Random forests. *Machine Learning* **45**: 5–32. DOI:10.1023/A:1010933404324.

Brevik EC, Cerdà A, Mataix-Solera J, Pereg L, Quinton JN, Six J, Van Oost K. 2015. The interdisciplinary nature of soil. *The Soil* **1**: 117–129. DOI:10.5194/soil-1-117-2015.

Brown L, Foster G. 1987. Storm erosivity using idealized intensity distribution. *American Society of Agricultural Engineers* **30**: 379–386.

Cai QG, Wang H, Curtin D, Zhu Y. 2005. Evaluation of the EUROSEM model with single event data on steeplands in the Three Gorges Reservoir Areas, China. *Catena* **59**: 19–23. DOI:10.1016/j.catena.2004.05.008.

Cammeraat LH. 2004. Scale dependent thresholds in hydrological erosion response of a semi-arid catchment in southeast Spain. *Agricultural Ecosystems and Environment* **104**: 317–332. DOI:10.1016/j.agee.2004.01.032.

Campbell GB. 1991. Soil physics with basic transport models for soil–plant systems, 1st edn. Elsevier: Amsterdam.

Cantón Y, Solé-Benet A, De Vente J, Boix-Fayos C, Calvo-Cases A, Asensio C, Puigdefábregas J. 2011. A review of soil erosion across scales in semi-arid south-eastern Spain. *Journal of Arid Environments* **75**: 1254–1261. DOI:10.1016/j.jaridenv.2011.03.004.

Capolongo D, Pennetta L, Piccarreta M, Fallacara G, Boenzi F. 2008. Spatial and temporal variations in soil erosion and deposition due to land-levelling in a semi-arid area of Basilicata (southern Italy). *Earth Surface Processes and Landforms* **33**: 364–379. DOI:10.1002/esp.1560.

Certini G, Vestgarden LS, Forte C, Tau SL. 2015. Litter decomposition rate and soil organic matter quality in a patchwork heathland of southern Norway. *The Soil* **1**: 207–2016. DOI:10.5194/soil-1-207-2015.

CMA. 2012. Chinese meteorological administration, Beijing: Climate Data. [http://2011.cma.gov.cn/en/; accessed: February 2014]

Copyright © 2016 John Wiley & Sons, Ltd.

1099145x, 2017, 4, Downloaded from https://onlinelibrary.wiley.com/doi/10.1002/ldr.2503 by alexandre Wadoux - Bibliothèque de Sorbonne Université , Wiley Online Library on [22/01/2025]. See the Terms and Conditions (https://onlinelibrary.wiley.com/terms-and-conditions) on Wiley Online Library for rules of use; OA articles are governed by the applicable Creative Commons License

De Vente J, Poesen J, Verstraeten G, Govers G, Vanmaercke M, Van Rompaey A, Arabkhedri M, Boix-Fayos C. 2013. Predicting soil erosion and sediment yield at regional scales: where do we stand? *Earth-Science Reviews* **127**: 16–29. DOI:10.1016/j.earscirev.2013.08.014.

De Vente J, Poesen J. 2005. Predicting soil erosion and sediment yield at the basin scale: scale issues and semi-quantitative models. *Earth-Science Reviews* **71**: 95–125. DOI:10.1016/j.earscirev.2005.02.002.

De Vente J, Poesen J, Arabkhedri M, Verstraeten G. 2007. The sediment yield problem revisited. *Progress in Physical Geography* **31**: 155–178. DOI:10.1177/0309133307076485.

Díaz-Uriarte R, De Andrés S. 2006. Gene selection and classification of microarray data using random forest. *BMC Bioinformatics* **7**: 3. DOI:10.1186/1471-2105-7-3.

Dunkerley D. 2008. Rain event properties in nature and in rainfall simulation experiments: a comparative review with recommendations for increasingly systematic study and reporting. *Hydrological Processes* **22**: 4415–4435. DOI:10.1002/hyp.7045.

Fang NF, Shi ZH, Yue BJ, Wang L. 2013. The characteristics of extreme erosion events in a small mountainous watershed. *PLoS One* **8**: 1–10. DOI:10.1371/journal.pone.0076610.

Ferreira V, Panagopoulos T, Andrade R, Guerrero C, Loures L. 2015. Spatial variability of soil properties and soil erodibility in the alqueva reservoir watershed. *Solid Earth* **6**: 383–392. DOI:10.5194/se-6-383-2015.

Flanagan DC, Nearing MA. 2000. Sediment particle sorting on hillslope profiles in the WEPP model. *American Society of Agricultural Engineers* **43**: 576–583.

Fryirs K. 2013. (Dis) connectivity in catchment sediment cascades: a fresh look at the sediment delivery problem. *Earth Surface Processes and Landforms* **38**: 30–46. DOI:10.1002/esp.3242.

Fuka DR, Walter MT, Archibald JA, Steenhuis TS, Easton ZM. 2014. 'EcoHydRology': community modeling foundation for Eco-hydrology. R package version 04-12. [http://cran.r-project.org/package=EcoHydRology; accessed: April 2014]

Gao ZQ, Zhang HJ, Shi YH. 2004. Study on soil erosion rate in different land use types in the granite area of three gorges. *Journal of Soil Water Conservation* **2**: 26–29.

García-Ruiz JM, Beguería S, Nadal-Romero E, Gonzáles-Hidalgo JC, Lana-Renault N, Sanjuán Y. 2015. A meta-analysis of soil erosion rates across the world. *Geomorphology* **239**: 160–173. DOI:10.1016/j.geomorph.2015.03.008.

Goebes P, Schmidt K, Seitz S, Stumpf F, Scholten T. 2015. Rule-based analysis of throughfall kinetic energy to evaluate leaf and tree architectural trait thresholds to mitigate erosive power. [accepted for publication in Progress in Physical Geography].

Green WH, Ampt GA. 1911. Studies on soil physics: I. The flow of air and water through soils. *Journal of Agricultural Sciences* **4**: 1–24.

Grønsten HA, Lundekvam H. 2006. Prediction of surface runoff and soil loss in southeastern Norway using the WEPP hillslope model. *Soil & Tillage Research* **85**: 186–199. DOI:10.1016/j.still.2005.01.008.

Gumiere SJ, Raclot D, Cheviron B, Davy G, Louchart X, Fabre J, Moussa R, Le Bissonais Y. 2011. MHYDAS-erosion: a distributed single-storm water erosion model for agricultural catchments. *Hydrological Processes* **25**: 1717–1728. DOI:10.1002/hyp.7931.

Guo Q, Hao Y, Liu B. 2015. Rates of soil erosion in China : a study based on runoff plot data. *Catena* **124**: 68–76. DOI:10.1016/j.catena.2014.08.013.

He X, Xu Y, Zhang X. 2007. Traditional farming system for soil conservation on slope farmland in southwestern China. *Soil & Tillage Research* **94**: 193–200. DOI:10.1016/j.still.2006.07.017.

Heung B, Bulmer CE, Schmidt MG. 2014. Predictive soil parent material mapping at a regional-scale: a random forest approach. *Geoderma* **214**: 141–154. DOI:10.1016/j.geoderma.2013.09.016.

Hill RD, Peart MR. 1998. Land use, runoff, erosion and their control: a review for southern China. *Hydrological Processes* **12**: 2029–2042. DOI:10.1002/(SICI)1099-1085(19981030)12:13/14<2029::AID-HYP717>3.0.CO;2-O.

Hooke J. 2003. Coarse sediment connectivity in river channel systems; a conceptual framework and methodology. *Geomorphology* **56**: 79–94.

Huang L, Ding SW, Zhang G, Peng YX. 1998. Preliminary estimation of soil and water conservation under different tillages on purple soil slope land in The Three Gorges Reservoir Area. *Journal of Huazhong Agricultural University* **17**: 45–49. [in Chinese]

Jetten VG, de Roo APJ, Favis-Mortlock D. 1999. Evaluation of field-scale and catchment-scale soil erosion models. *Catena* **3**: 521–541. DOI:10.1016/S0341-8162(99)00037-5.

Jetten V, Govers G, Hessel R. 2003. Erosion models: quality of spatial predictions. *Hydrological Processes* **17**: 887–900. DOI:10.1002/hyp.1168.

Jordan G, Van Rompaey A, Szilassi P, Csillag G, Mannaerts C, Woldai T. 2005. Historical land use changes and their impact on sediment fluxes in the Balaton basin (Hungary). *Agriculture, Ecosystems & Environment* **108**: 119–133. DOI:10.1016/j.agee.2005.01.013.

Kepa Brian Morgan TK, Sardelic DN, Waretini AF. 2012. The Three Gorges Project: how sustainable? *Journal of Hydrology* **460**: 1–12. DOI:10.1016/j.jhydrol.2012.05.008.

Keesstra SD, Geissen V, Van Schaik L, Mosse K, Piiranen S. 2012. Soil as a filter for groundwater quality. *Current Opinions in Environmental Sustainability* **4**: 507–516. DOI:10.1016/j.cosust.2012.10.007.

Keesstra SD, Bruijnzeel LA, Van Huissteden J. 2009. Meso-scale catchment sediment budgets: combining field surveys and modeling in the dragonja catchment, southwest Slovenia. *Earth Surface Processes and Landforms* **34**: 1547–1561. DOI:10.1002/esp.1846.

Kirkby MJ. 1978. Hillslope hydrology. Wiley & Sons Ltd: Chichester.

Krause P, Boyle DP, Bäse F. 2005. Comparison of different efficiency criteria for hydrological model assessment. *Advances in Geosciences* **5**: 89–97. DOI: hal-00296842.

Lal R. 2003. Soil erosion and the global carbon budget. *Environment International* **29**: 437–450. DOI:10.1016/S0160-4120(02)00192-7.

Lee G, Yu W, Jung K. 2013. Catchment-scale soil erosion and sediment yield simulation using a spatially distributed erosion model. *Environmental Earth Sciences* **70**: 33–47. DOI:10.1007/s12665-012-2101-5.

Lesschen JP, Schoorl JM, Cammeraat LH. 2009. Modelling runoff and erosion for semi-arid catchment using a multi-scale approach based on hydrological connectivity. *Geomorphology* **109**: 174–183. DOI:10.1016/j.geomorph.2009.02.030.

Lexartza-Artza I, Wainwright J. 2011. Making connections: changing sediment sources and sinks in an upland catchment. *Earth Surface Processes and Landforms* **36**: 1090–1104. DOI:10.1002/esp.2134.

Liaw A, Wiener M. 2002. Classification and regression by random forest. *R News* **2**: 18–22.

Liu J, Liu M, Tian H, Zhuang D, Zhang Z, Zhang W, Tang X, Deng X. 2005. Spatial and temporal patterns of China's cropland during 1990–2000: an analysis based on Landsat TM data. *Remote Sensing Environment* **98**: 442–456. DOI:10.1016/j.rse.2005.08.012.

López-Vicente M, Navas A. 2010. Routing runoff and soil particles in a distributed model with GIS: implications for soil protection in mountain agricultural landscapes. *Land Degradation & Development* **21**: 100–109. DOI:10.1002/ldr.901.

López-Vicente M, Poesen J, Navas A, Gaspar L. 2013. Predicting runoff and sediment connectivity and soil erosion by water for different land use scenarios in the Spanish Pre-pyrenees. *Catena* **102**: 62–73. DOI:10.1016/j.catena.2011.01.001.

López-Vicente M, Quijano M, Palazón L, Gaspar L, Navas A. 2015. Assessment of soil redistribution at catchment scale by coupling a soil erosion model and a sediment connectivity index (central Spanish Pre-Pyrenees). *Cuadernos de Investigación Geográfica* **41**: 127–147. DOI: 10.18172/cig.2649.

Lu XX, Higgitt DL. 2000. Estimating erosion rates on sloping agricultural land in the Yangtze Three Gorges, China, from caesium-137 measurements. *Catena* **39**: 33–51. DOI:10.1016/S0341-8162(99)00081-8.

Marchamalo M, Hooke JM, Sandercock PJ. 2015. Flow and sediment connectivity in semi-arid landscapes in SE Spain: patterns and control. *Land Degradation & Development* . DOI:10.1002/ldr.2352.

Martínez-Mena M, Albaladejo J, Castillo VM. 1998. Factors influencing surface runoff generation in a Mediterranean semi-arid environment. *Hydrological Processes* **12**: 74–754.

McBratney AB, Mendonça Santos ML, Minasny B. 2003. On digital soil mapping. *Geoderma* **117**: 3–52. DOI:10.1016/S0016-7061(03)00223-4.

Merz J, Dangol PM, Dhakal MP, Dongol BS, Nakarmi G, Weingartner R. 2006. Rainfall-runoff events in a middle mountain catchment of Nepal. *Journal of Hydrology* **331**: 446–458. DOI:10.1016/j.jhydrol.2006.05.030.

Michael A. 2000. Anwendung des physikalisch begründeten Erosionsprognosemodells EROSION 2D/3D - Empirische Ansätze zur Ableitung der Modellparameter. Technical University Freiberg, Germany. [Dissertation]

Mitasova H, Hofierka J, Zlocha M, Iverson LR. 1996. Modeling topographic potential for erosion and deposition using GIS. *International Journal of Geographic Information Systems* **10**: 629–641. DOI:10.1080/02693799608902101.

Morgan RPC. 2005. Soil erosion and conservation. Blackwell Publishing: Oxford.

Nash JE, Sutcliff JV. 1970. River flow forecasting through conceptual model. Part 1: a discussion of principles. *Journal of Hydrology* **10**: 282–290. DOI:10.1016/0022-1694(70)90255-6.

Copyright © 2016 John Wiley & Sons, Ltd.

1099145x, 2017, 4, Downloaded from https://onlinelibrary.wiley.com/doi/10.1002/ldr.2503 by alexandre Wadoux - Bibliothèque de Sorbonne Université , Wiley Online Library on [22/01/2025]. See the Terms and Conditions (https://onlinelibrary.wiley.com/terms-and-conditions) on Wiley Online Library for rules of use; OA articles are governed by the applicable Creative Commons License

Nathan RJ, McMahon TA. 1990. Evaluation of automated techniques for base flow and recession analysis. *Water Resources Research* **26**: 1465–1473. DOI:10.1029/WR026i007p01465.

Nearing MA, Govers G, Darrell NL. 1999. Variability in soil erosion data from replicated plots. *Soil Science Society of America Journal* **63**: 1829–1835. DOI:10.2136/sssaj1999.6361829x.

Nearing MA. 2000. Evaluating soil erosion models using measured plot data: accounting for variability in the data. *Earth Surface Processes and Landforms* **25**: 1035–1043.

Nearing MA, Jetten V, Baffaut C, Cerdan O, Couturier A, Hernandez M, Le Bissonnais Y, Nichols MH, Nunes JP, Renschler CS, Souchère V, Van Oost K. 2005. Modeling response of soil erosion and runoff to changes in precipitation and cover. *Catena* **61**: 131–154. DOI:10.1016/j.catena.2005.03.007.

Onyando JO, Kisoyan P, Chemelil MC. 2005. Estimation of potential soil erosion for river Perkerra catchment in Kenya. *Water Resources Management* **19**: 133–143. DOI:10.1007/s11269-005-2706-5.

Palazón L, Gaspar L, Latorre B, Blake WH, Navas A. 2014. Evaluating the importance of surface soil contributions to reservoir sediment in alpine environments: a combined modelling and fingerprinting approach in the Posets-Maladeta Natural Park. *Solid Earth* **5**: 963–978. DOI:10.5194/se-5-963-2014.

Park S, Oh C, Jeon S, Jung H, Choi C. 2011. Soil erosion risk in Korean watersheds, assessed using the revised universal soil loss equation. *Journal of Hydrology* **399**: 263–273. DOI:10.1016/j.jhydrol.2011.01.004.

Peng CUI, Yonggang GE, Yongming LIN. 2011. Soil erosion and sediment control effects in the Three Gorges Reservoir Region, China. *Journal of Resources and Ecology* **2**: 289–297. DOI:10.3969/j.issn.1674-764x.2011.04.001.

Peters J, Baets BD, Verhoest NEC, Samson R, Degroeve S, Becker PD, Huybrechts W. 2007. Random forests as a tool for ecohydrological distribution modelling. *Ecological Modelling* **207**: 304–318. DOI:10.1016/j.ecolmodel.2007.05.011.

Puigdefábregas J. 2005. The role of vegetation patterns in structuring runoff and sediment fluxes in drylands. *Earth Surface Processes and Landforms* **30**: 133–148. DOI:10.1002/esp.1181.

Quine TA, Walling DE, Chakela QK, Mandiringana OT, Zhang X. 1999. Rates and patterns of tillage and water erosion on terraces and contour strips: evidence from caesium-137 measurements. *Catena* **36**: 115–142. DOI:10.1016/S0341-8162(99)00006-5.

RapidEye. 2012. Satellite imagery product specifications—version 4.1. [http://www.rapideye.com/upload; accessed: February 2013]

Renard K, Foster G, Weesies G, McCool D, Yoder D. 1997. Predicting soil erosion by water: a guide to conservation planning with the revised universal soil loss equation (RUSLE). USDA agricultural handbook No. 703. [http://www.osmre.gov/resources/library/ghm/USDA_AH703.pdf; accessed: May 2014]

Rickemann D, McArdell BW. 2007. Continuous measurement of sediment transport in the Erlenbach stream using piezoelectric bedload impact sensors. *Earth Surface Processes and Landforms* **32**: 1362–1378. DOI:10.1002/esp.1478.

SAGA. 2012. System for automated geoscientific analyses -version 2.0.6. [http//:www.saga-gis.org; accessed: January 2013]

Satterland DR, Adams PW. 1992. Wildland watershed management. Wiley & Sons Ltd: New Jersey.

Schindewolf M, Schmidt J. 2012. Parameterization of the EROSION 2D/3D soil erosion model using a small-scale rainfall simulator and upstream runoff simulation. *Catena* **91**: 47–55. DOI:10.1016/j.catena.2011.01.007.

Schmidt J. 1991. A mathematical model to simulate rainfall erosion. *Catena* Supplememt **19**: 101–109.

Schmidt J. 1992. Modeling long term soil loss and landform change. In: Parson AJ, Abrahams AD (Eds.). Overland flow — hydraulics and erosion mechanics. UCL Press, London.

Schmidt J, Werner MV, Michael A. 1999. Application of the EROSION 3D model to the CATSOP watershed, the Netherlands. *Catena* **37**: 449–456. DOI:10.1016/S0341-8162(99)00032-6.

Schönbrodt S, Saumer P, Behrens T, Seeber C, Scholten T. 2010. Assessing the USLE crop and management factor C for soil erosion modelling in a large mountainous watershed in central China. *Journal of Earth Science* **21**: 835–845. DOI:10.1007/s12583-010-0135-8.

Schönbrodt-Stitt S, Bosch A, Behrens T, Hartmann H, Shi X, Scholten T. 2013a. Approximation and spatial regionalization of rainfall erosivity based on sparse data in a mountainous catchment of the Yangtze river in central China. *Environmental Science and Pollution Research* **20**: 6917–6933. DOI:10.1007/s11356-012-1441-8.

Schönbrodt-Stitt S, Behrens T, Schmidt K, Shi X, Scholten T. 2013b. Degradation of cultivated bench terraces in the Three Gorges Area: Field mapping and data mining. *Ecological Indicators* **34**: 478–493. DOI:10.1016/j.ecolind.2013.06.010.

Shen ZY, Gong YW, Li YH, Hong Q, Xu L, Liu RM. 2009. A comparison of WEPP and SWAT for modeling soil erosion of the Zhangjiachong Watershed in the Three Gorges Reservoir Area. *Agricultural Water Management* **96**: 1435–1442. DOI:10.1016/j.agwat.2009.04.017.

Shen ZY, Gong YW, Li YH, Liu RM. 2010. Analysis and modeling of soil conservation measures in the Three Gorges Reservoir Area in China. *Catena* **81**: 104–112. DOI:10.1016/j.catena.2010.01.009.

Shi ZH, Cai CF, Ding SW, Wang TW, Chow TL. 2004. Soil conservation planning at the small watershed level using RUSLE with GIS: a case study in the Three Gorge Area of China. *Catena* **55**: 33–48. DOI:10.1016/S0341-8162(03)00088-2.

Shi ZH, Ai L, Fang NF, Zhu HD. 2012. Modeling the impacts of integrated small watershed management on soil erosion and sediment delivery: a case study in the Three Gorges Area, China. *Journal of Hydrology* **438**: 156–167. DOI:10.1016/j.jhydrol.2012.03.016.

Slimane AB, Raclot D, Evrad O, Sanaa M, Lefevre I, Bissonais YL. 2015. Relative contributions of rill/interrill and gully/channel erosion to small reservoir siltation in Mediterranean environments. *Land Degradation & Development* . DOI:10.1002/ldr.2387.

Soulis KX, Valiantzas JD, Dercas N, Londra PA. 2009. Analysis of the runoff generation mechanism for the investigation of the SCS-CN method applicability to a partial area experimental watershed. *Hydrological Earth System Sciences* **6**: 373–400. DOI:10.5194/hess-13-605-2009.

Starkloff T, Stolte J. 2014. Applied comparison of the erosion risk models EROSION 3D and LISEM for a small catchment in Norway. *Catena* **118**: 154–167. DOI:10.1016/j.catena.2014.02.004.

Strehmel A, Schönbrodt-Stitt S, Buzzo G, Dumperth C, Stumpf F, Zimmermann K, Bieger K, Behrens T, Schmidt K, Bi R, Rohn J, Hill J, Udelhoven T, Xiang W, Shi X, Cai Q, Jiang T, Fohrer N, Scholten T. 2015. Assessment of geo-hazards in a rapidly changing landscape: the three Gorges Reservoir Region in China. *Environmental Earth Sciences* **74**: 4939–4960. DOI:10.1007/s12665-015-4503-7.

Stumpf F, Schmidt K, Behrens T, Schönbrodt-Stitt S, Buzzo G, Dumperth C, Wadoux A, Xiang W, Scholten T. 2015a. Incorporating limited field operability and legacy soil samples in a hypercube sampling design for digital soil mapping. [under review]

Stumpf F, Schmidt K, Goebes P, Behrens T, Schönbrodt-Stitt S, Wadoux A, Xiang W, Scholten T. 2015b. Spatial uncertainty-guided sampling to improve digital soil maps. [under review]

Taguas EV, Moral C, Ayuso JL, Pérez R, Gómez JA. 2011. Modeling the spatial distribution of water erosion within a Spanish olive orchard microcatchment using the SEDD model. *Geomorphology* **133**: 47–56. DOI:10.1016/j.geomorph.2011.06.018.

Takken I, Beuselinck L, Nachtergaele J, Govers G, Poesen J, Degraer G. 1999. Spatial evaluation of a physically-based distributed erosion model (LISEM). *Catena* **37**: 431–447. DOI:10.1016/S0341-8162(99)00031-4.

Tan Y, Yao F. 2006. Three gorges project: effects of resettlement on the environment in the reservoir area and countermeasures. *Population and Environment* **27**: 351–371. DOI:10.1007/s11111-006-0027-0.

Tarboton DG. 1997. A new method for the determination of flow directions and upslope areas in grid digital elevation models. *Water Resources Research* **33**: 309–319. DOI:10.1029/96WR03137.

Terranova O, Antronico L, Coscarelli R, Iaquinta P. 2009. Soil erosion risk scenarios in the Mediterranean environment using RUSLE and GIS: an application model for Calabria (southern Italy). *Geomorphology* **112**: 228–245. DOI:10.1016/j.geomorph.2009.06.009.

Todisco F. 2014. The internal structure of erosive and non-erosive storm events for interpretation of erosive processes and rainfall simulation. *Journal of Hydrology* **519**: 3651–3663. DOI:10.1016/j.jhydrol.2014.11.002.

Verheijen FGA, Jones RJA, Rickson RJ, Smith CJ. 2009. Tolerable versus actual soil erosion rates in Europe. *Earth-Science Reviews* **94**: 23–38. DOI:10.1016/j.earscirev.2009.02.003.

Verstraeten G, Poesen J, Gillijns K, Govers G. 2006. The use of riparian vegetated filter strips to reduce river sediment loads: an overestimated control measure? *Hydrological Processes* **20**: 4259–4267. DOI:10.1002/hyp.6155.

Vanmaercke M, Poesen J, Verstraeten G, De Vente J, Ocakoglu F. 2011. Sediment yield in Europe: spatial patterns and scale dependency. *Geomorphology* **130**: 142–161. DOI:10.1016/j.geomorph.2011.03.010.

Copyright © 2016 John Wiley & Sons, Ltd.

1099145x, 2017, 4, Downloaded from https://onlinelibrary.wiley.com/doi/10.1002/ldr.2503 by alexandre Wadoux - Bibliothèque de Sorbonne Université , Wiley Online Library on [22/01/2025]. See the Terms and Conditions (https://onlinelibrary.wiley.com/terms-and-conditions) on Wiley Online Library for rules of use; OA articles are governed by the applicable Creative Commons License

Van Rompaey A, Verstraeten G, Van Oost K, Govers G, Poesen J. 2001. Modelling mean annual sediment yield using a distributed approach. *Earth Surface Processes and Landforms* **26**: 1221–1236.

Van Rompaey A, Govers G. 2002. Data quality and model complexity for continental scale soil erosion modelling. *International Journal of Geographical Information Science* **16**: 663–680. DOI:10.1080/13658810210148561.

Weigert A, Schmidt J. 2005. Water transport under winter conditions. *Catena* **64**: 193–208. DOI:10.1016/j.catena.2005.08.009.

Willmott CJ, Matsuura K. 2005. Advantages of the mean absolute error (MAE) over the root mean square error (RMSE) in assessing average model performance. *Climate Research* **30**: 79–82.

Wischmeier WH, Smith DD. 1981. Predicting rainfall erosion losses – a guide to conservation planning. U.S. Department of Agriculture, Handbook 537. [http://naldc.nal.usda.gov/download/CAT79706928/PDF; accessed: April 2014]

WRB. 2014. World reference base for soil resources. [http://www.fao.org/soils-portal/soil-survey/soil-classification/world-reference-base/en/; accessed: March 2015]

Wu DM, Yu YC, Xia LZ, Yin SX, Yang LZ. 2011. Soil fertility indices of citrus orchard land along topographic gradients in the three gorges area of China. *Pedosphere* **21**: 782–792. DOI:10.1016/S1002-0160(11)60182-3.

Xiang WS, Liang CF, Li WH. 2001. Soil and water loss from cultivated slope land derived from granite under different cropping systems in the Three Gorges Reservoir area. *Chinese Journal of Applied Ecology* **12**: 47–50. [in Chinese]

Xie Y, Liu B, Nearing MA. 2002. Practical thresholds for separating erosive and non-erosive events. *American Society of Agricultural Engineers* **45**: 1843–1847.

Xu X, Tan Y, Yang G, Li H, Su W. 2011. Impacts of China's Three Gorges Dam Project on net primary productivity in the reservoir area. *Science of the Total Environment* **409**: 4656–4662. DOI:10.1016/j.scitotenv.2011.08.004.

Xu X, Tan Y, Yang G. 2013. Environmental impact assessments of the Three Gorges Project in China: issues and interventions. *Earth-Science Reviews* **124**: 115–125. DOI:10.1016/j.earscirev.2013.05.007.

Zambrano-Bigiarini M. 2014. 'hydroGOF': goodness-of-fit functions for comparison of simulated and observed hydrological time series. R package version 0.3-8. [https://cran.r-project.org/web/packages/hydroGOF/index.html; accessed: July 2015].

Zhao G, Mu X, Wen Z, Wang F, Gao P. 2013. Soil erosion, conservation, and eco-environment changes in the Loess Plateau of China. *Land Degradation & Development* **24**: 499–510. DOI:10.1002/ldr.2246.

Zhang B. 2008. Impact on mountainous agricultural development in the Three Gorges Reservoir Area forced by migrants of the Three Gorges Project. *Chinese Journal of Population Resources and Environment* **6**: 83–89. DOI:10.1080/10042857.2008.10684886.

Zhang L, O'Neill AL, Lacey S. 1996. Modelling approaches to the prediction of soil erosion in catchments. *Environmental Software* **11**: 123–133. DOI:10.1016/S0266-9838(96)00023-8.

Zhang J, Zhengjun L, Xiaoxia S. 2009. Changing landscape in the Three Gorges Reservoir Area of Yangtze River from 1977 to 2005: Land use/land cover, vegetation cover changes estimated using multi-source satellite data. *International Journal of Applied Earth Observation and Geoinformation* **11**: 403–412. DOI:10.1016/j.jag.2009.07.004.

Zhang Q, Lou Z. 2011. The environmental changes and mitigation actions in the Three Gorges Reservoir region, China. *Environmental Science & Policy* **14**: 1132–1138. DOI:10.1016/j.envsci.2011.07.008.

Zheng WW, Zhang CL. 2006. Soil conservation benefit analysis for citrus orchard in the reservoir basin of Three Gorges. *Subtropical Soil Water Conservation* **18**: 15–18.

Zhou P, Luukkanen O, Tokola T, Nieminen J. 2008. Effect of vegetation cover on soil erosion in a mountainous watershed. *Catena* **75**: 319–325. DOI:10.1016/j.catena.2008.07.010.

Zhu AX, Liu J, Du F, Zhang SJ, Qin CZ, Burt J, Behrens T, Scholten T. 2015. Predictive soil mapping with limited sample data. *European Journal of Soil Science* **66**: 535–547. DOI:10.1111/ejss.12244.

Copyright © 2016 John Wiley & Sons, Ltd.

1099145x, 2017, 4, Downloaded from https://onlinelibrary.wiley.com/doi/10.1002/ldr.2503 by alexandre Wadoux - Bibliothèque de Sorbonne Université , Wiley Online Library on [22/01/2025]. See the Terms and Conditions (https://onlinelibrary.wiley.com/terms-and-conditions) on Wiley Online Library for rules of use; OA articles are governed by the applicable Creative Commons License
